# Reconciling Gene Tree Discordance and Biogeography in European Crows

**DOI:** 10.1111/mec.17764

**Published:** 2025-04-10

**Authors:** Chyi Yin Gwee, Dirk Metzler, Jérôme Fuchs, Jochen B. W. Wolf

**Affiliations:** ^1^ Division of Evolutionary Biology LMU Munich Planegg‐Martinsried Germany; ^2^ Microevolution and Biodiversity Max Planck Institute for Biological Intelligence Seewiesen Germany; ^3^ Institut de Systématique, Evolution, Biodiversité (ISYEB), CNRS, SU, EPHE, UA Muséum National d'Histoire Naturelle Paris France

**Keywords:** gene tree, genomic landscape, introgression, islands of differentiation, isolation‐with‐migration, suture zone

## Abstract

Reconstructing the evolutionary history of young lineages diverging with gene flow is challenging due to factors like incomplete lineage sorting, introgression, and selection causing gene tree discordance. The European crow hybrid zone between all‐black carrion crows and grey‐coated hooded crows exemplifies this challenge. Most of the genome in Western and Central European carrion crow populations is near‐identical to hooded crows, but differs substantially from their Iberian congeners. A notable exception is a single major‐effect colour‐locus under sexual selection aligning with the ‘species’ tree. To understand the underlying evolutionary processes, we reconstructed the biogeographic history of the species complex. During the Pleistocene carrion and hooded crows took refuge in the Iberian Peninsula and the Middle East, respectively. Allele‐sharing of all‐black Western European populations with likewise black Iberian crows at the colour‐locus represents the last trace of carrion crow ancestry, resisting gene flow from expanding hooded crow populations that have homogenised most of the genome. A model of colour‐locus introgression from an Iberian ancestor into hooded crow populations near the Pyrenées was significantly less supported. We found no positive relationship between introgression and recombination rate consistent with the absence of genome‐wide, polygenic barriers in this young species complex. Overall, this study portrays a scenario where few large‐effect loci, subject to divergent sexual selection, resist rampant and asymmetric gene exchange. This study underscores the importance of integrating population demography and biogeography to accurately interpret patterns of gene tree discordance following population divergence.

## 1 Introduction

The widespread availability of genomic data has advanced our ability to infer the relationships among organisms at both macro‐ (Jiang et al. 2020; Kong et al. 2022; Song et al. 2012; Xi et al. 2014) and micro‐evolutionary levels (Bourgeois and Warren 2021; Ellegren 2014; Patterson et al. 2012; Wohns et al. 2022). However, resolving evolutionary relationships is often complicated by conflicting gene trees shaped by evolutionary processes such as incomplete lineage sorting (ILS), gene flow, and selection. These processes can manifest as reticulated phylogeny (Maddison 1997) or as landscapes of heterogenous genomic differentiation (Ravinet et al. 2017; Wolf and Ellegren 2017). To overcome this challenge, multispecies coalescent models have been applied to reconstruct single‐species trees (Degnan 2018; Edwards et al. 2016; Hibbins et al. 2023; Mirarab et al. 2021), and a wide range of approaches have been developed to infer ancestry and demographic histories (Bourgeois and Warren 2021; Durand et al. 2011; Loog 2020; Moorjani and Hellenthal 2023; Patterson et al. 2012). Recent studies have revealed that this discordance among gene trees is not merely an inconvenience, but could reflect biologically meaningful variation in genealogies and ultimately provide information on population ancestry, admixture, demographic changes, and selection (Durand et al. 2011; Edelman et al. 2019; Martin and Van Belleghem 2017; Patterson et al. 2012). It remains a key challenge in evolutionary biology to extract the relevant information for understanding the demographic and selective processes involved in adaptation and speciation (Brauer et al. 2023; Enbody et al. 2023; Fontaine et al. 2015; Fu and Akey 2013; Racimo et al. 2015; Suvorov et al. 2022; Svardal et al. 2020).

**FIGURE 1:**
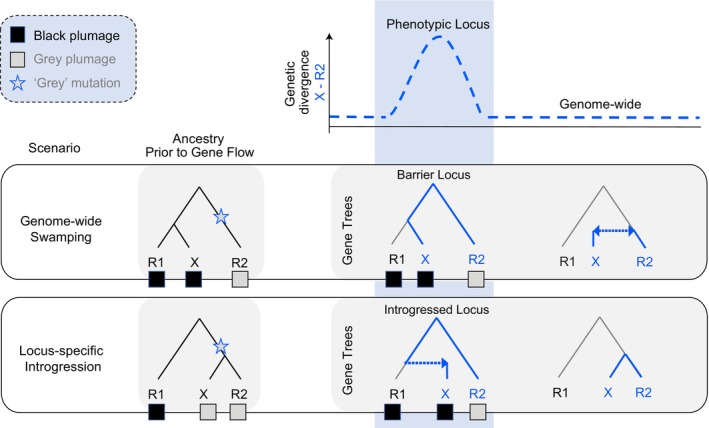
Schematic representation of gene trees for three populations under two different evolutionary scenarios. We consider two refugial populations (R1, R2) exchanging genes with a third population (*X*) sharing original ancestry with either R1 or R2. Effective migration between population *X* and either of the refugial populations is indicated with blue arrows. Gene trees are shown for a locus coding for phenotypic variation (black/grey plumage) and an unlinked, neutrally evolving locus from elsewhere in the genome. Genetic divergence is measured between populations R2 and *X* and is equally elevated for the ‘phenotypic locus’ (illustrated in blue) in both scenarios giving rise to the same genomic landscape of differentiation. Under the ‘genome‐wide swamping’ scenario, gene flow homogenises R2 and *X* and distorts ancestry relationships genome‐wide. The phenotypic locus is maintained by divergent selection (barrier locus) and represents the ‘species tree’ prior to gene flow. By contrast, under a scenario of ‘locus‐specific introgression’, gene trees generally reflect the ‘species tree’ prior to gene flow which is only distorted in the phenotypic locus by introgression from population R1.

Young rapidly radiating groups are particularly susceptible to gene tree discordance (Lopes et al. 2021; Meyer et al. 2016). Not only is ILS a common issue as insufficient time has passed for ancestral polymorphisms to sort (Maddison 1997), ongoing gene flow is rampant among recently diverging lineages in overlapping ranges (Payseur and Rieseberg 2016). In the early stages of population divergence, alleles of neutrally evolving genomic regions can freely move between populations, whereas effective migration is reduced in regions subject to divergent selection (barrier loci) (Barton and Bengtsson 1986; Wu 2001). This process ultimately results in a gene tree mosaic across the genome with islands of increased genetic differences between populations against a backdrop of low genetic differentiation at unlinked neutrally evolving genomic regions (Jones et al. 2012; Ravinet et al. 2017; Wolf and Ellegren 2017). However, the same heterogeneous gene tree pattern can arise by adaptive introgression, in which selectively advantageous regions of a source population can leak into the gene pool of another population (Edelman et al. 2019; Mérot et al. 2013; The Heliconius Genome Consortium 2012). An ‘aberrant’ gene tree can thus be attributed to fundamentally different processes: it could either represent a barrier locus under divergent selection resisting ‘genomic swamping’ by an invading population or, on the contrary, reflect ‘locus‐specific introgression’ into a heterospecific background (see Figure 1).

It is, therefore, crucial to distinguish between the two scenarios to identify the underlying mechanisms causing gene tree discordance and to determine their roles in neutral evolution, adaptation and/or speciation (Cruickshank and Hahn 2014; Edelman and Mallet 2021; Ravinet et al. 2017; Wolf and Ellegren 2017). Cline analysis is frequently applied to infer barrier strength between hybridising lineages and to disentangle neutral processes from adaptive introgression (Barton and Hewitt 1985; Cruzan et al. 2021; Stankowski et al. 2017; Szymura and Barton 1986). In the absence of selection, symmetric gene flow of neutrally evolving loci widens the cline with the centre remaining close to the contact zone (Barton and Hewitt 1985). In the presence of divergent selection, a sharp cline is maintained at the contact zone at barrier loci. Adaptive introgression will similarly leave a sharp locus‐specific cline, but shift the cline centre towards the recipient of beneficial alleles (Cruzan et al. 2021). An adaptive introgression cline can, therefore, be mistaken for one resulting from divergent selection, and distinguishing between the two is non‐trivial. In both cases, a phenotypic association is expected, which can be assessed through methods such as quantitative trait loci (QTL) and genome‐wide association scanning (GWAS) (Fontaine et al. 2015; Racimo et al. 2015; Whitney et al. 2015). In the case of adaptive introgression, signatures of positive selection are expected (Sankararaman et al. 2014; Vernot and Akey 2014), but overlapping processes such as linked selection can complicate inference (Burri 2017; Burri et al. 2015), necessitating a thorough understanding of demographic history (Mattila et al. 2019; Wall et al. 2002).

An important component to distinguish the scenarios is to infer population history from neutral genetic variation. As population dynamics do not take place in a void, reconstruction can be greatly aided by integrating demographic reconstruction with biogeographic background information: global climatic oscillations during the Pleistocene entail inescapable changes in species distributions with periods of isolation and periods of opportunity for introgression. Independent observations from different species contextualise genomic variation within a spatio‐temporal framework under which divergent traits may have evolved. Suture zones that assort non‐randomly in space provide historical evidence for this phenomenon where organisms expand out of their refugia and come into secondary contact in these regions (Hewitt 1988, 2001; Remington 1968). In Europe, several suture zones have been identified, as illustrated by the contemporary distributions of three well‐studied species: the meadow grasshopper, *Pseudochorthippus parallelus* (Butlin and Hewitt 1985; Cooper et al. 1995; Hagberg et al. 2022), the European hedgehog, *Erinaceus europeus* (Bolfíková and Hulva 2012; Santucci et al. 1998; Seddon et al. 2002), and the brown bear, *Ursus arctos* (Taberlet and Bouvet 1997; Waits et al. 2000). For each of these suture zones, mountain ranges such as the Pyrenées and Alps act as physical barriers to the expansion of refugial populations from Iberia, Italy, the Balkans, and/or Caucasus (Hewitt 2000, 2001). Interestingly, these mountain ranges influence the expansion routes of the refugial populations differently, resulting in slight variations in the contemporary distributions of populations and the locations of hybrid zones (Hewitt 1999; Taberlet et al. 1998).

**FIGURE 2:**
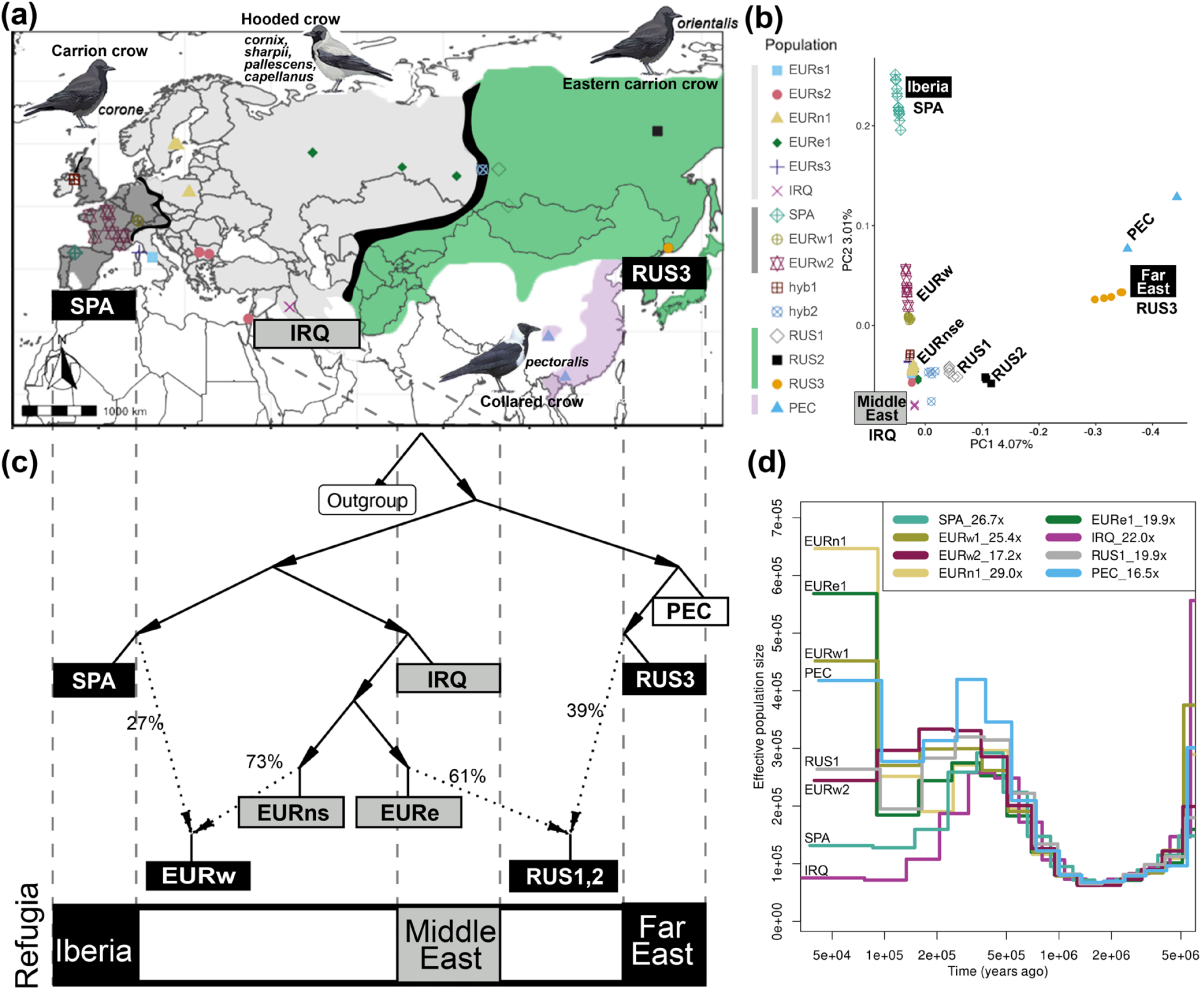
Population structure of the *Corvus corone* species complex. Colour codes and shapes used for individual populations are standardised throughout the figure. (a) Geographic representation of the species complex and sample localities used in this study, including carrion crow *corone* (SPA_in_, EURw_est_), hooded crow *cornix* (EURn_orth_, EURe_ast_), *sharpii/pallescens* (EURs_outh_), *capellanus* (IR_a_Q), eastern carrion crow *orientalis* (RUS_sia_1‐3), and collared crow *pectoralis* (PEC). Background colours of the map correspond to the distribution of carrion crow (dark grey), hooded crow (light grey), eastern carrion crow (green) and collared crow (purple). Hybrid zones are each demarcated by black lines, and the closest descendants of potential refugial populations are labelled in squares at the bottom of the map. (b) Principal Component Analysis (PCA) based on 14.8 M variants. (c) Admixture graph illustrating genome‐wide population relationships including two admixture events inferred across the European and Siberian hybrid zone. Within Europe, the Spanish (SPA) and Iraqi (IRQ) crow populations are found to be most diverged, and are referred to as the Iberian and Middle Eastern refugia, respectively. Colours reflect plumage phenotype. (d) Effective population size of each population inferred by MSMC2 with a representative genome of at least 15x coverage (individual coverage shown in the legend).

The *Corvus corone* species complex is a classic example of a hybrid zone, illustrating an extreme case of gene tree discordance (Poelstra et al. 2014; Vijay et al. 2016). The plumage patterns of this species complex follow a leap‐frog pattern, with an all‐black phenotype in Western and Central Europe (*C. (c.) corone*) and Siberia (*C. (c.) orientalis*), intersected by a grey‐coated phenotype across Northern, Eastern, and Southern Europe (*C*. *(c.) cornix, C. (c.) sharpii, C. (c.) pallescens*, and *C. (c.) capellanus*), as well as a similar pied phenotype in Central Asia (*C. (c.) pectoralis*) (see Figure 2a) (for subspecies delineation see Parkin et al. 2003; for taxonomic treatment see Vijay et al. 2016). In Europe, all‐black carrion crows and grey‐coated hooded crows form a narrow morphological hybrid zone (Meise 1928). Assortative mating and marginalisation of minority phenotypes have been repeatedly documented (Brodin and Haas 2009; Meise 1928; Randler 2007; Rolando 1993; Saino 1991) and appear to be sufficient for long‐term stable maintenance of a phenotypic cline (Metzler et al. 2021). Hybrids are fertile and there is no evidence for intrinsic post‐zygotic isolation (Saino and Villa 1992). Despite the presumed stability of the hybrid zone, the allelic composition of the all‐black Western and Central European carrion crow is near‐identical to that of the grey‐coated hooded crows. Only a small proportion of the genome (< 1%) conforms to a gene tree separating all‐black Western and Central European crows from hooded crow populations (Poelstra et al. 2014; Vijay et al. 2016). This aberrant pattern is dominated by a major‐effect locus spanning a ~2 Mb region on chromosome 18 (chr18). This region contains pigmentation genes that, together with the gene *NDP* on chr1, explain most of the variation in plumage coloration (Knief et al. 2019). Important for the context of this study, alleles of the gene on chr18 and chr1 interact non‐additively. Model simulations suggest that epistatic interaction between loci can induce hybrid zone movement (Metzler et al. 2021), implying that the initial contact zone between the black and grey‐coated crows may have been displaced through time. Under such a scenario, the contemporary morphological hybrid zone, which shows steep divergence for the colour loci, would differ from the location of the original zone of contact.

The contemporary morphological hybrid zone places the point of contact in Central Europe, suggesting a post‐glacial migration route akin to the brown bear, *U. arctos* (Taberlet and Bouvet 1997; Waits et al. 2000). On the contrary, the genomic similarities of Western European carrion crows and hooded crows may hint at a different contact zone in the Pyrenées, akin to the meadow grasshopper, *P. parallelus* (Butlin and Hewitt 1985; Cooper et al. 1995; Hagberg et al. 2022) and several bird lineages, including *Picus viridis* /*sharpei* (Pons et al. 2011, 2019) and *Muscicapa striata* (Pons et al. 2016). These scenarios have fundamentally different implications for the interpretation of selection. In the first scenario, secondary contact of carrion and hooded crow ancestries would have occurred in Central Europe. The lack of genome‐wide divergence would result from rampant and asymmetric gene flow from hooded crows into carrion crow ancestry—resisted only by few barrier loci coding for plumage. Gene tree discordance in this case would arise from divergent selection maintaining ancestral divergence against gene flow across the rest of the genome. The second scenario, on the contrary, implies secondary contact occurred west of the current hybrid zone, along the Pyrenées, followed by locus‐specific introgression of Iberian ‘black pigmentation’ alleles into an ancestral hooded crow genomic background. Here, gene tree discordance would be due to gene flow at single colour loci, while the majority of the genome would retain its original ancestry, unaffected by introgression.

Here, we study gene tree discordance in the context of spatial, temporal, and demographic dynamics of a broad population sample, including key populations from candidate past refugia and regions of secondary contact. First, we provide a broad overview of admixture history across the entire species complex. We then focus on the European hybrid zone, specifically contrasting a ‘genome‐wide swamping’ scenario with a ‘locus‐specific introgression’ scenario using tree‐based approaches and site frequency spectrum‐based coalescent simulations. By interpreting demographic history in the context of Pleistocene biogeography, our study provides spatially explicit insights into the interplay between gene flow and selection, resulting in a mosaic genomic landscape that aligns with a morphologically stable hybrid zone.

## 2 Methods

### 2.1 Population Sampling

Whole‐genome sequencing data were obtained from a total of 135 individuals of the *C. corone* species complex, including population samples of Central Asian *C. (c.) pectoralis*, Siberian *C. (c.) orientalis*, European *C. (c.) corone*, *C. (c.) cornix*, *C. (c.) sharpii*, *C. (c.) pallescens*, *and C. (c.) capellanus*. Of these, sequence data for 118 individuals were obtained from earlier studies (Poelstra et al. 2014; Vijay et al. 2016). Sequence data for 17 individuals were newly generated in this study, including tissues of *C. (c.) corone* from France (*n* = 9), feathers of *C. (c.) cornix* from Corse (*n* = 3), and tissues of the Mesopotamian crow *C. (c.) capellanus* from Iraq (*n* = 5), which has the palest plumage among all subspecies of the grey‐coated hooded crow (see Figure S1). Further details on all samples, including accession numbers of sequencing data stored in the Sequencing Read Archive (SRA) of the National Center for Biotechnology (NCBI), are provided in Table S1.

### 2.2 Raw Data Processing

Raw reads were treated with Cutadapt 1.18 (Martin 2011) to remove flanking adapters and reassessed with FastQC (Andrews 2010). The trimmed reads were mapped to the hooded crow reference genome (GenBank accession no. GCA_000738735.1) with BWA 0.7.17 (Li 2013) and converted from SAM to BAM format using SAMtools 1.7 (Li et al. 2009). Read group information was added and duplicates were removed using picard 2.25.7 (http://broadinstitute.github.io/picard).

DNA damage was assessed with MapDamage 2.0 (Jónsson et al. 2013) and was detected in the toe pad samples of *C. (c.) pectoralis* with elevated levels of C to T and G to A deamination for the 5′ and 3′ ends of reads. The two toe pad samples were further trimmed to remove 5 bp from the beginning of each forward and reverse read. Sample Un_un_P01 was ultimately removed as deamination persisted when reassessed with mapDamage 2.0.

Variant calling was conducted using three callers: ANGSD 0.933 (Korneliussen et al. 2014); BCFtools 1.10.2 (Danecek et al. 2021); and GATK 4.2.6.1 (McKenna et al. 2010). In particular, GATK's HaplotypeCaller was used to generate both variant and invariant sites, allowing the inclusion of invariant site information for specific analyses, such as computing summary statistics of *d*_XY_ and the site frequency spectrum (SFS), with monomorphic sites accounted for (see Supporting Information Text for the parameters applied for each caller and the quality filtering process). Overall, we found that 82.1% of the variants were identified by all three callers (Figure S2). A total of 14,935,463 biallelic single nucleotide polymorphisms (SNPs) remained after low‐quality variants, indels, and repeated regions were removed with BCFtools. The one‐dimensional SFS of each population was examined and an excess of variants at frequency 0.5 was observed, suggesting the presence of paralogs. Variant sites that are not on chr18 and significantly differ from Hardy–Weinberg equilibrium were identified with VCFtools 0.1.14 (Danecek et al. 2011) and omitted, thus removing the potential paralogs. A final set of 14,822,221 biallelic SNPs was retained for downstream analysis.

### 2.3 Exploratory Analysis on Population Structure

Principal component analysis (PCA) of the *C. corone* species complex was computed with SNPRelate 1.20.0 (Zheng et al. 2012) on the final set of 14,822,221 SNPs to assess population structure. ADMIXTURE 1.3.0 (Alexander et al. 2009) was conducted to estimate individual ancestries using maximum likelihood for up to 10 clusters. Before running ADMIXTURE, the variants were processed to remove linkage disequilibrium with PLINK 1.90b6.21 (Purcell et al. 2007). Linkage disequilibrium was estimated using a window size of 25 variants, a step size of 10 variants and an *R*‐squared threshold of 0.1 (‐indep‐pairwise 25 10 0.1). A total of 7.7 M unlinked biallelic SNPs remained, which were used to conduct ADMIXTURE and another PCA to assess any difference in the reduced SNP set.

Summary statistics, including nucleotide diversity (π) and pairwise genetic differentiation (*F*_ST_ and *d*_XY_) were calculated using the script popgenwindows.py (see https://github.com/simonhmartin/genomics_general) in 50,000 bp window along the genome. The final set of biallelic SNPs was concatenated with invariant sites called by GATK using BCFtools to generate a VCF containing both variant and invariant sites for computation of summary statistics. Additionally, net genetic divergence (*d*_a_), which represents the number of taxon‐specific mutations accumulated since the divergence of the most recent common ancestor, was calculated using the formula *d*_*a*_ = *d*_XY_ – (π_*x*_ + π_*y*_)/2. Scaffolds were mapped to chromosomes using the chromosomal hooded crow reference genome (GenBank accession no. GCA_000738735.5).

### 2.4 Admixture Graph and Detecting Introgression

Admixture among different populations of the *C. corone* species complex was examined with ADMIXTOOLS2 (Maier et al. 2023), an R package adapted from ADMIXTOOLS (Patterson et al. 2012), which uses *f*_4_‐statistics to infer the proportion of admixture. We used two alternative outgroups in each run: the closely related *C. brachyrhynchos* and the more distantly related *C. moneduloides* . This approach allowed us to assess how the choice of outgroup might affect the results. To generate comparable sites for the outgroup, six individuals of *C. brachyrhynchos* and five individuals of *C. moneduloides* were mapped to the same hooded crow reference genome. The raw reads of these 11 samples were processed following the raw data processing pipeline outlined above. All sites were genotyped using GATK 3.8.1, then merged with the VCF containing 14.8 M biallelic SNPs across the *C. corone* complex using BCFtools. Only biallelic variant sites with no missing individuals were retained for the analysis. The ‘find graphs’ function was run to obtain the best‐fitting admixture graph with zero to two admixture events. Each run consists of a maximum of 1,000 generations of trees, which terminated when the likelihood score did not improve after 50 generations of trees with random pruning committed to the last best tree. The process was repeated 100 times to ensure that the sampling space was not constrained by the starting tree.

Dsuite (Malinsky et al. 2021) was used to compute *D*‐statistics and *f*_4_‐ratios (Patterson et al. 2012) of all possible combinations of three populations and an outgroup using the option ‘Dtrios’. The generated *D*‐statistics and *f*_4_‐ratios detect excess sharing of alleles between two populations (ABBA > BABA) and estimate ancestry proportions of an admixed proportion, respectively. The option ‘Fbranch’ was subsequently applied on a predefined input tree with the output of Dtrios to estimate f‐branch statistics for each branch on the tree, including internal branches, to estimate admixture at different time periods.

### 2.5 Effective Population Sizes Through Time

Effective population size (Ne) through time was estimated using three different methods: (i) Multiple Sequentially Markovian Coalescent (MSMC2) (Schiffels and Durbin 2014; Schiffels and Wang 2020), a coalescent with recombination approximation approach; (ii) SMC++ 1.15.2 (Terhorst et al. 2017), a recombination and SFS‐based approach; and (iii) Stairway Plot 2 (Liu and Fu 2015, 2020), a SFS approach. Present‐day effective population size of selected populations was also estimated using fastsimcoal2 (Excoffier et al. 2021) and Jaatha 3.2.5 (Mathew et al. 2013; Naduvilezhath et al. 2011) (see *Demographic inference* below). For all subsequent demographic analyses, a generation time of 5.79 years and a mutation rate of 3.18 × 10^−9^ per generation were used (Vijay et al. 2016).

In brief, we used a genome of at least 15× coverage per population for the MSMC analysis. Only the largest 100 scaffolds from macrochromosomes without previous evidence for divergent selection (i.e., chr1A, and chr1‐5) were considered for both the MSMC2 and SMC++ analysis. For Stairway Plot analysis, we used 5,774,276 unlinked and putatively neutral SNPs (see *Neutral sites generation* below) to obtain the folded SFS of each population with easySFS (Gutenkunst et al. 2009). The length of the genome was scaled to 329,623,861 bp to account for unlinked and neutral regions using the following formula: number of SNPs used/total neutral SNPs × (total neutral SNPs + total neutral invariant sites). For more detail, see Supporting Information Text.

### 2.6 Neutral Sites Generation

We used the python script vcfsummarizer.py (https://github.com/emjosephs/popGen/blob/master/vcfSummarizer.py) to identify non‐coding regions, including 4‐fold degenerate, intronic, and intergenic variant sites from the final set of 14.8 M SNPs for each population (Kutschera et al. 2020). The annotation was based on NCBI *Corvus cornix* Annotation Release 100 (assembly accession number: GCF_000738735.1; Poelstra et al. 2014). These sites were combined across all populations, and scaffolds of chr18 were removed. A total of 11,146,221 putatively neutral SNPs and 5,774,276 putatively neutral and unlinked SNPs were generated for demographic analyses. The same script was applied to the invariant sites generated by GATK described previously (see raw data processing above) to obtain the number of comparable ‘neutral’ invariant sites (*n* = 625,134,484, excluding chr18) for demographic scaling.

### 2.7 Demographic Inference

Fastsimcoal 2.7 (Excoffier et al. 2021) and Jaatha 3.2.5 (Mathew et al. 2013; Naduvilezhath et al. 2011) were used to infer whether the neutrally evolving regions of the genome better conforms to the ‘genome‐wide swamping’ scenario or the ‘locus‐specific introgression’ scenario (Figure 1). We simulated datasets for both scenarios including descendants of the two allopatric, putative refugial populations (SPA, IRQ), and admixed populations adjacent to the current hybrid zone (EURw vs. EURn & EURs). The hooded crows from Northern and Southern Europe were collapsed into a single population (henceforth EURns) as they show little genetic differentiation and are likely panmictic. The ‘genome‐wide swamping’ scenario assumes shared ancestry between ‘refugial’ populations and geographically adjacent populations sharing the same morphology ((SPA, EURw), (IRQ, EURns)). The ‘locus‐specific introgression’ scenario assumes a common ancestor for hooded crow populations and the Western European carrion crow populations separated from the Iberian refugium ((IRQ, (EURw, EURns)), SPA). We used 11,146,221 putatively neutral SNPs to generate folded joint‐SFS (j‐SFS) of the six population pairs with easySFS (Gutenkunst et al. 2009). We randomly selected 15 diploid individuals from each population, except for the Iraqi population, where all five individuals were included. In both models, we allowed population growth for the Western European and North‐southern European crows as suggested by a negative Tajima's value of around −1 (Table S2). Below is a brief outline of both approaches (refer to Supporting Information Text for a detailed description, Figure S3 for a comparison of both methods, and Table S3 for the parameter ranges used).

#### 2.7.1 Fastsimcoal

For each model, 100 independent replicates were conducted with fastsimcoal 2.7. Each replicate involved 1,000,000 coalescent simulations per cycle to estimate the expected folded j‐SFS and 100 Expectation‐Conditional Maximisation (ECM) (Meng and Rubin 1993) cycles to estimate the model parameters. We selected the best‐fitting estimates of each model based on the replicate with the lowest difference between the observed and expected maximum likelihoods. The best‐fitting estimates of each model were compared against each other on the basis of the Akaike information criterion (AIC) (Bozdogan 1987). AIC was computed as AIC = 2 *k*—2ln(log_10_(*L*)), where k is the number of parameters and log_10_(*L*) is the log10 likelihood reported by fastsimcoal. Parametric bootstrap was conducted on the best‐fitting model by simulating 100 independent j‐SFS. Each j‐SFS was generated with 636,281 independent loci of 1000 bp with a transition rate of 0.5 (i.e., transition/transversion ratio of 2), under a finite‐sites mutation model. The parameters for each of the 100 simulated j‐SFS were re‐estimated with 1,000,000 coalescent simulations and 100 ECM cycles. The initial parameter values for these estimations were set to the best‐fitting model, allowing for the computation and maximisation of the composite likelihood for each simulated SFS.

#### 2.7.2 Jaatha

We additionally ran the composite‐likelihood optimisation method Jaatha 3.2.5 for model choice and estimation of demographic parameters. The j‐SFS of each population pair was segmented into 14 bins of polymorphism to reduce computational complexity. Jaatha can simultaneously infer recombination rates, which could potentially improve the accuracy of demographic parameter estimations. Statistics based on violations of the four‐gamete condition (Hudson and Kaplan 1985) in pairs of polymorphic sites were applied to infer recombination. We calculated the fraction of sites violating the four‐gamete condition in 91 putatively neutral and randomly chosen sets of 10,000 bp DNA sequences from 15 SPA, 24 EURw, 54 EURns, and five IRQ each. The R package scrm (Staab et al. 2015) was used to simulate 91 loci of 10,000 bp with recombination under an infinite sites model to estimate the expected values for the four‐gamete condition‐based statistics.

To simulate j‐SFS with 400 loci of 2000 bp without recombination, Jaatha was run with msprime (Baumdicker et al. 2022) for each cycle using an infinite sites model. The number of loci and sequence length were selected based on previous experiments with Jaatha and msprime to balance accuracy and computation efficiency. In each iteration step of Jaatha, 1000 such simulations were performed. The zoom‐in method was applied and repeated five times for the initial search of parameter values. Parametric bootstrap was conducted by launching 100 simulations of j‐SFS with the fitted parameters using both infinite and finite site models to assess possible bias. The simulated SFS were generated with 636,281 loci of 1000 bp using msprime to scale to neutral genome size (see Supporting Information Text). For the finite‐sites bootstrap simulations, we combined msprime and scrm with seq‐gen (Rambaut and Grassly 1997) and applied a HKY model with a transition/transversion ratio of 2 (Hasegawa et al. 1985). The bootstrap simulations were repeated with fastsimcoal and vice versa to assess model fitness with a different simulator. Re‐estimations of the model parameters were conducted with the same procedure as described above. Bootstrap bias correction (Efron and Tibshirani 1994) was conducted and quantiles were calculated with default settings of the quantile function in R.

### 2.8 Gene Tree Inference

We constructed gene trees along the genome using 'Topology weighting by iterative sampling of trees' (Twisst) (Martin and Van Belleghem 2017; Van Belleghem et al. 2017). In brief, this method constructs subtrees by iteratively sampling an individual from each taxon and conducts weighting of all subtrees for the possible topologies (e.g., three possible topologies among three ingroup taxa). We ran Twisst with three (SPA, EURw1, and EURn1/IRQ) and four populations (SPA, EURw1, EURn1, and IRQ), using *C. moneduloides* as an outgroup in each run. Gene trees were generated following the recommended pipeline, in which the genome was first phased using beagle 5.4 (Browning et al. 2018). Gene trees were generated in blocks of 50 SNPs with neighbour joining and GTR model for a total of 287,217 blocks of genomic regions, of which 285,323 blocks do not contain the aberrant locus from chr18. Additionally, we ran Twisst on the trees simulated by the parameter values estimated from the best run of each of the two demographic models with fastsimcoal. An outgroup was also included in the simulation with an effective population size of 10,000 and a divergence time of 10 M years from the most recent common ancestor of IRQ and SPA. A total of 285,323 independent trees, each generated from 2500 bp non‐recombining DNA sequence, were simulated with an average of 49 to 53 SNPs per block. The choice of a 2500 bp sequence length was made to generate approximately 50 SNPs per block, ensuring comparability with the empirical approach above, which used 50 SNPs for each gene tree. The simulated trees were then used as input for the Twisst analysis as described above. Ternary plots were generated using ggtern 3.5.0 (Hamilton and Ferry 2018) on R 4.4.1 to examine the distribution of topology weighting. To assess the simulated and empirical distribution of subtree topologies, the sites were grouped into 400 bins using the function ‘stat_tri_bin’ on ggtern and the difference in sites count of each bin was computed between each of the simulated model and the empirical data.

### 2.9 Introgression and Recombination Rate

We used chromosome length as an approximation for the recombination rate (*r*). In birds, chromosome length is a well‐established predictor of recombination rate, varying over several orders of magnitude across chromosomes (Singhal et al. 2015). To assess if this negative correlation holds true in the crow system, we calculated the population recombination rate *ρ* (=4*N*_*e*_ *r*) in 50,000 bp windows, following Vijay et al. (2016), for the Spanish population, which is least affected by gene flow. We find a strong negative correlation between the mean recombination rate against chromosome length (*r* = −0.87, *p* = 0.000).

To estimate the degree of introgression, we calculated the chromosome‐average of the *f*_dM_ statistic estimated in windows of 50 SNPs (Malinsky et al. 2021). Genome‐wide introgression was estimated between EURw and EURn using both refugial populations (SPA and IRQ) as alternative references: ((SPA,EURw),EURn),outgroup) and (((IRQ,EURn),EURw),outgroup). In both cases, a positive f_dM_ value indicates elevated gene flow between EURw and EURn.

## 3 Results

### 3.1 Population Structure of the Species Complex Across Eurasia

We re‐evaluated the population structure of the *C. corone* species complex, adding hitherto unsampled populations from areas of candidate Pleistocene refugia and secondary contact in the Western Palearctic. Overall, the complex shows a weak isolation‐by‐distance pattern across the Palearctic, with all‐black plumage crows from Spain (SPA; *corone*) and the Far East (RUS3; *orientalis*) displaying the endpoints of genetic variance along PC1 (Figure 2b) (see also Vijay et al. 2016). Important for the context of this study, *C*. (*c.) capellanus* from Iraq (IRQ) is genomically distinct from the otherwise remarkably homogenous hooded crows, including *C. (c.) cornix*, *C. (c.) sharpii*, and *C. (c.) pallescens*, across Northern, Southern, and Eastern Europe (EURnse) (Figure 2b, Figure S4, Table S4). The average *F*_ST_ of all pairwise comparisons among EURnse, excluding the islandic Corse crows (EURs3), was six times lower (*F*_ST_ = 0.009) than the average *F*_ST_ of IRQ with any of the EURnse populations (*F*_ST_ = 0.059). Among all pairwise comparisons of carrion and hooded crow populations (Table S4), IRQ has the highest mean pairwise genetic differentiation with SPA (*F*_ST_ = 0.0903) which is consistent with the largest distance on PC2. Therefore, SPA and IRQ may represent the closest descendants of Pleistocene refugia in the Iberian Peninsula and Middle East, respectively.

Admixture graph illustrates a history of massive admixture within the species complex, specifically between populations of contrasting plumage types across both the European and Siberian hybrid zones (Figure 2c, Figure S5). The western Siberian population of all‐black *orientalis* crows (RUS1,2) is highly admixed, sharing the majority (61%) of genomic ancestry with grey‐coated crows (EURe1), but only 39% with all‐black RUS3. Similarly, the all‐black Western European crows (EURw), ranging from the French Pyrenees to Germany, are highly admixed. They share 73% of genomic ancestry with grey‐coated crows from Northern and Southern Europe (EURns), but only 27% with all‐black SPA (Figure 2c). This result is largely consistent with inferred ancestry contributions using ADMIXTURE and f‐branch statistics (Figures S6 and S7).

Additionally, MSMC2 analysis shows that SPA and IRQ have the smallest effective population sizes among carrion and hooded crows (Figure 2d). Consistently, SMC++ and Stairway plot2 results indicate a decrease in effective population size for SPA and IRQ around 20,000 years ago (kya) (Figure S8), suggesting that these populations may represent the closest descendants of Pleistocene refugia.

Overall, the genome‐wide ancestry of several members of the species complex is inconsistent with their phenotypes. Despite similar levels of admixture proportion in the European and Siberian hybrid zones, they do not share the same genomic landscape of differentiation between populations from the hybrid zone (Poelstra et al. 2014; Vijay et al. 2016). A previous study has demonstrated that the genomic landscape of differentiation between populations from the Siberian hybrid zone does not show a single highly differentiated locus, but multiple smaller peaks (Vijay et al. 2016). In contrast, comparisons between populations from each side of the European hybrid zone predominantly identify a single aberrant locus on chr18 remaining highly differentiated in both contact and non‐contact zones (Vijay et al. 2016; Figures S9 and S10).

Genome‐wide divergence of hooded crow populations (reported here EURn1), measured by mean *F*_ST_, is lowest with the adjacent population from Germany (EURw1, *F*_ST_ = 0.0119), slightly elevated with the population from France (EURw2, *F*_ST_ = 0.0160), and shows a sharp increase with the population from Spain (SPA; *F*_ST_ = 0.0743). Overall, the genetic similarity of carrion crows to the putative refugial descendants from Spain increases with geographical proximity (Figure S4b). These results are consistent with ‘genome‐wide swamping’, suggesting massive gene flow extending across the morphological hybrid zone, resisted by chr18 and halted genome‐wide at the boundary to the Pyrenées. However, the results are also consistent with the ‘locus‐specific introgression’ scenario, assuming EURw and EURns share recent ancestry, while allelic variation on chr18 spread into France with slight genome‐wide spillover at the Pyrenées boundary.

### 3.2 Demography of the European Crow Complex

**FIGURE 3:**
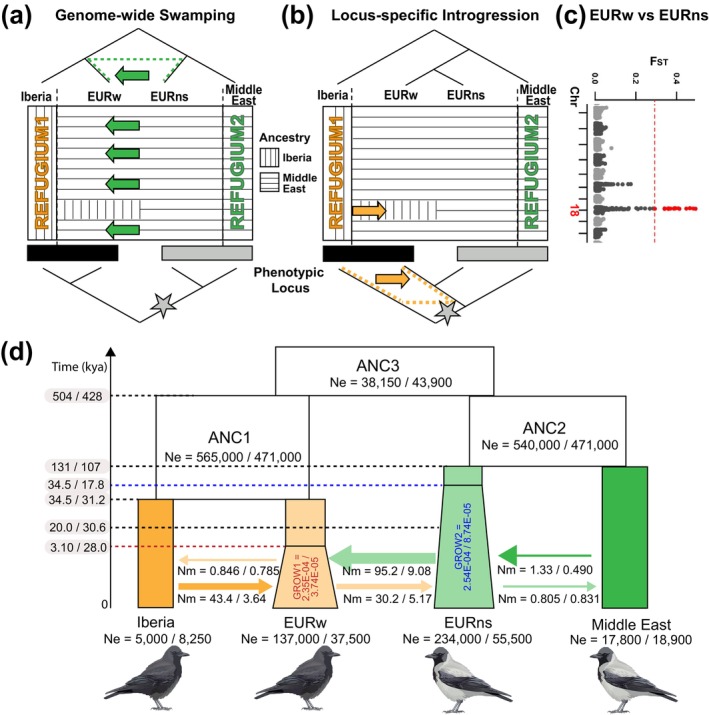
Comparison of the ‘genome‐wide swamping’ and ‘locus‐specific introgression’ models. (a–c) Schematic representation of the evolutionary history under both scenarios. The cladogram at the top shows the true ancestries prior to gene flow. The cladogram at the bottom illustrates the ancestral relationships at the locus coding for plumage colouration (all‐black or grey‐coated), with the grey mutation marked by a star. Gene flow is shown with green and orange dotted lines. The box in the middle places population ancestry in a biogeographic and spatial context. In both scenarios, SPA and IRQ represent descendants of Iberian and Middle Eastern refugia, respectively and are least affected by gene flow. (a) The ‘genome‐wide swamping’ scenario assumes shared ancestry between ‘refugial’ populations and geographically adjacent populations sharing the same morphology. Asymmetric genome‐wide gene flow (green arrows) across a zone of secondary contact in central Europe homogenises EURw and EURns reducing genome‐wide differentiation (*F*_ST_) to near‐zero (panel c). The phenotypic locus is maintained by divergent selection (barrier locus) and represents the ‘species tree’ prior to gene flow as indicated by high genetic differentiation (panel c; outlier locus indicated by red points on chr18). (b) The ‘locus‐specific introgression scenario, assumes that Western European carrion crows share the most recent common ancestor with hooded crows rather than with the Iberian carrion crows. A zone of secondary contact west of the current hybrid zone provides an opportunity for introgression of black alleles on chr18 into Western Europe (orange arrow) but does not affect shared recent ancestry with hooded crows genome‐wide (panel c). (d) Demographic modelling using fastsimcoal and Jaatha provides evidence that the ‘genome‐wide swamping’ model fits the data better. Western European populations migrated out of Iberia during the Last Glacial and experienced high amount of gene flow from the Northern and Southern European population upon secondary contact. Parameter values reflect estimates from fastsimcoal/Jaatha. ANC, ancestral population; Ne, effective population size; Nm, number of migrants per generation.

To investigate the underlying process leading to the single aberrant locus observed in all‐black EURw, we contrasted two demographic models referring to ‘genome‐wide swamping’ or ‘locus‐specific introgression’ (Figure 3a–c). Due to sample constraints in the Eastern Palearctic, we focused on European populations in candidate past refugia of carrion (SPA) and hooded crows (IRQ), and admixed populations in proximity to the current hybrid zone (EURw, EURns). We report parameter estimates for the model of each scenario from two different approaches, fastsimcoal and Jaatha. The values are presented in the same order, separated by a ‘/’, where appropriate.

For both fastsimcoal and Jaatha, the ‘genome‐wide swamping’ model fit the data better than the ‘locus‐specific introgression’ model based on likelihoods and overall fit to the observed joint site frequency spectrum (j‐SFS) (Figure 3d, Figures S11–S13, AIC: 430,169,400 vs. 430,364,442/20,034,646 vs. 23,388,757). Assessment of model fit, by comparing the expected and observed j‐SFS simulated with both finite and infinite‐site models, as well as with and without bootstrap‐based bias correction, supports this result (Figures S13 and S14, Tables S5 and S6). The best parametrisation of the ‘genome‐wide swamping’ model fits the data well overall and consistently shows a relatively low number of over‐ or underestimated sites in the j‐SFS (Figures S11 and S12). Only sites of high frequency in EURw and low frequency in EURns are slightly underestimated (Figures S11 and S12, blue areas in j‐SFS of EURw and EURns), suggesting that some regions under selection and/or linked selection may be included in the putatively neutral dataset. We identified 293 underestimated sites, primarily from scaffold 7 (chr8; 161 sites) and scaffold 29 (chr15; 73 sites). These candidate regions contain ten genes, none of which, however, are known to be involved in the melanogenesis pathway (Table S7). The fit of the ‘locus‐specific introgression’ model was visibly poorer, particularly overestimating divergence between the two all‐black crow populations (Figures S11 and S12, extensive red areas in the j‐SFS of SPA and EURw).

According to parameter estimation of the best‐fit ‘genome‐wide swamping’ model, refugial populations from Iberia and Middle East (represented by SPA and IRQ, respectively) diverged around 504/428 kya (Figure 3d, Tables S5 and S6). This divergence estimate is comparable to indirect inference from MSMC2 (Figure 2d; 500 kya) and the net genetic divergence (*d*_a_) of SPA and IRQ populations on chr18 reflecting pre‐gene flow ancestry (Table S8; 343 kya). Hooded crows outside of IRQ (EURns) diverged from the Middle Eastern refugium 131/107 kya, followed by a split of EURw from the Iberian refugium 34.5/31.2 kya. Since the split, gene flow within the all‐black populations was slightly asymmetrical with more individuals migrating out of the refugial population from Iberia into Central Europe (migration rate per generation (*m*) = 3.16E‐04/9.70E‐05 of SPA to EURw vs. 1.69E‐04/9.52E‐05 of EURw to SPA). In contrast, gene flow between the Middle Eastern and grey‐coated populations was relatively low and symmetrical (*m* = 5.68E‐06/8.82E‐06 of IRQ to EURns vs. 4.52E‐05/4.41E‐05 of EURns to IRQ). Importantly, both models infer rampant gene flow between all‐black EURw and grey‐coated EURns since 20.0/30.6 kya, shortly after EURw separated from Iberia. Gene flow was highly asymmetric with comparatively more individuals migrating from EURns to EURw (*m* = 6.94E‐04/2.42E‐04 of EURns to EURw vs. 1.29E‐04/9.32E‐05 of EURw to EURns). This is consistent with an expanding wave of hooded crows into the territory of all‐black crows (cf. Figure 3a).

While the estimates of divergence time and migration rates are relatively similar between fastsimcoal and Jaatha, we found inconsistent estimates of growth rate per generation (GR) and time since expansion (TEGR) for EURw and EURns (Tables S5 and S6). Jaatha estimated EURw and EURns each grew since 28 and 17.8 kya, respectively, at similar rates (GR = 3.74E‐05 and 8.74E‐05, respectively). In contrast, fastsimcoal estimated a shorter period of growth for EURw and higher growth rates for both populations (TEGR = 3.10 and 34.5 kya, GR = 2.35E‐04 and 2.54E‐04 for EURw and EURns, respectively). Overall, the percentage increase in effective population size was comparable between the estimates by fastsimcoal and Jaatha due to the inverse relationship between the parameters TEGR and GR. Taken together, these results provide strong support for the scenario of genome‐wide swamping of the Iberian ancestry of EURw by asymmetric gene flow from grey‐coated EURns.

### 3.3 Gene Tree Discordance

**FIGURE 4:**
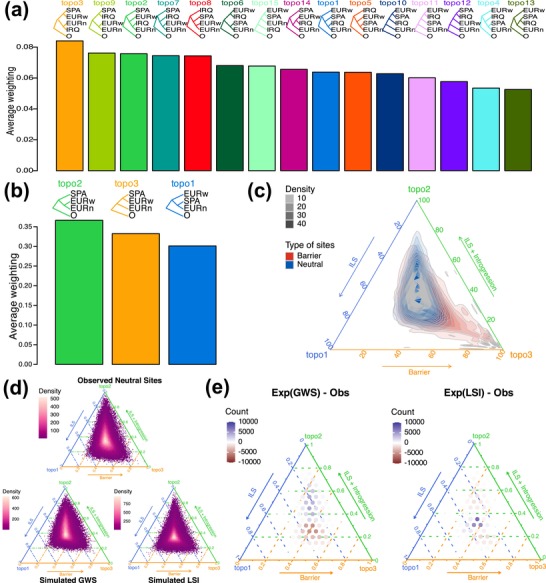
Gene tree‐based ancestry inference under high levels of introgression among recently diverging lineages. (a) Most subtrees generated by iterative sampling of four populations consisting of both refugial populations (IRQ, SPA) reflect the true ancestry of the Western European population (EURw) prior to gene flow (with and without the barrier sites from chr18, see Figure S15). (b) Most subtrees generated by iterative sampling of the three populations (SPA, EURw and EURn) show a topology of incomplete lineage sorting (ILS) and introgression (topo2) instead of ancestry prior to gene flow (topo3). (c) Ternary plot of the distribution of barrier and neutral sites with varying proportion of each of the three topologies. A two‐dimensional kernel density estimation was performed with a detection limit threshold of 0.005 for barrier and neutral sites separately. Neutral sites distribute around the ternary centroid with a low density of loci skewing towards topo2. The distribution of barrier sites skews to the right with a low density of loci reflecting topo3 exclusively. (d) Ternary plots of the distribution of neutral loci from the observed (Obs) data, and simulated from the fastsimcoal estimates for the ‘genome‐wide swamping’ (GWS) and ‘locus‐specific introgression’ (LSI) scenarios. Each plane is divided into 150 hexagonal bins. (e) Difference (computed in 400 bins) between the distribution of topologies from simulated and observed data for the GWS and LSI model. Overestimated and underestimated bins are shown in blue and red, respectively.

Next, we examined the effect of population sampling on tree‐based ancestry inference by conducting iterative sampling of subtrees of three to four populations, consisting of SPA, EURw, EURn, and IRQ. The four‐population analysis shows that most subtrees generated from all variant sites support a closer ancestry between EURw and SPA, in agreement with the demographic inference (Figure 4a). Most of the subtrees show the correct ancestry with and without the inclusion of the aberrant locus on chr18 (Figure S15). Therefore, iterative sampling of subtrees, in this case appears capable of distinguishing a scenario of recent ancestry between EURw and EURn (‘locus‐specifc introgression’) from a scenario of ancestral divergence homogenised by recent gene flow (‘genome‐wide swamping’).

Contrary to the results of four‐population trees (Figure 4a) and simulations (Figure 3d), the three‐population analysis using SPA and the two contact zone populations (EURw and EURn) supports a closer ancestry between EURw and EURn than between EURw and SPA (Figure 4b). Most loci have a near‐equal proportion of all topologies indicative of ILS and provide little ancestral information (Figure 4c, Figure S16a; sites clustering in the centroid of the ternary plot). Variant sites showing a clear topological preference provide information about introgression and barriers to gene flow (Figure 4c, Figure S16a; sites at each tip of the ternary plot). An excess of loci showing preference for topo2 over topo1 and topo3 reflects introgression between EURw and EURn (Figure S16a; more sites skewing towards topo2 than topo3). Predictably, loci at and above the 99.9th percentile of subtrees reflecting topo3 are primarily found on chr18, especially in scaffold 78, where 100% of the subtrees depict topo3 (Figure 4c, Figure S16b,c). On the contrary, the three‐population analysis with IRQ replacing EURn shows that most subtrees support the correct ancestry prior to gene flow (Figure S16d). Sites with a high proportion of subtrees reflecting the correct ancestry are also found throughout the genome, not only on chr18, potentially due to a low level of introgression between EURw and IRQ (Figure S16e). These results highlight the importance of population sampling in discerning ancestral history.

Last, we integrated the framework of gene tree topologies with demographic simulations for putatively neutrally evolving sites. We explored how well gene tree topologies simulated under the best parameterisations of the ‘genome‐wide swamping’ and ‘locus‐specific introgression’ models in fastsimcoal fit the empirical data. Consistent with the empirical results for three populations (SPA, EURw and EURn), simulated trees under both models primarily reflect ILS (equal proportion of topo1‐3, Figure 4d) and produce subtrees that predominantly support a closer ancestry between EURw and EURns (skew towards topo2, Figure 4d, Figure S17). The ‘locus‐specific introgression’ model deviates from the empirical data with an overestimation of uninformative ILS loci, depicting all three topologies in equal proportions (Figure 4e: dark blue circles on the centroid). The ‘genome‐wide swamping’ model differs less from the empirical gene tree distribution, but slightly overestimates gene flow, as indicated by an excess of loci supporting a grouping of EURw and EURn (Figure 4e: excess topo2, shown in blue) and a corresponding depletion of loci supporting the other two topologies (Figure 4e; red circles skewing below the centroid). Simulated trees of both models for four‐population analysis, including IRQ, predominantly support a closer ancestry between EURw and SPA, in agreement with the empirical results (Figure S18). The predominant topology of ((SPA,EURw),(EURns,IRQ)) over (SPA,(IRQ,(EURns,EURw))) in the ‘locus‐specific introgression’ model suggests that a simulated scenario with high gene flow between SPA and EURw could hypothetically lead to incorrect ancestry inference for the alternative model. Taken together, these results highlight the limitations of using gene trees to distinguish between genome‐wide swamping and shared recent ancestry, in particular if only one of the refugial populations was included. The findings thus underscore the importance of comprehensive population sampling and illustrate its diagnostic value in assessing model fit in demographic reconstruction.

### 3.4 Introgression and Recombination Rate

So far, we have contrasted locus‐specific introgression with genome‐wide introgression in putatively neutral regions, assuming a single migration rate across the genome. Yet, polygenic species barriers may introduce heterogeneity in the degree of introgression. Conventionally, introgression is expected to be more strongly impeded in regions of low recombination due to deleterious effects in hybrids, leading to an expected positive correlation between introgression and recombination rate and a negative correlation between introgression and chromosome length (Hennelly et al. 2021; Martin et al. 2019; Schumer et al. 2018). However, we found no significant relationship between the degree of introgression (*f*_dM_) and chromosome length, either with the Iberian (*p* = 0.094, *r* = 0.32) or the Middle Eastern (*p* = 0.257, *r* = 0.22) refugial populations as a reference (Figure S19). As expected, chr18 shows an exceptionally low level of introgression compared to other chromosomes of similar length.

## 4 Discussion

The evolutionary history of *C. corone* species complex is characterised by young population divergence accompanied by extensive gene flow (Kryukov et al. 2012; Vijay et al. 2016). While the Eastern Palearctic and Western Palearctic groups each form distinct clusters connected by an isolation‐by‐distance pattern, population ancestries are difficult to resolve by conventional tree‐based methods. Indeed, current and past studies have demonstrated that the complex forms a highly reticulated phylogenetic history, with the Western and Eastern populations of *orientalis* and the phenotypically defined *coron*e exhibiting paraphyly (Kryukov et al. 2012; Vijay et al. 2016). Using an admixture graph (Patterson et al. 2012; Pickrell and Pritchard 2012), the topology of the species complex is more accurately represented as a network, identifying European and Siberian populations surrounding current hybrid zones as groups of admixed ancestry. However, the proportion of genomic similarity with each ancestral group obtained from the contemporary population may not accurately reflect the population's ancestry prior to introgression, as selective forces can differentially affect neutral and barrier regions. To clarify evolutionary processes underlying gene tree discordance, we reconstructed the genome‐wide evolutionary history of the European part of the species complex to identify its ancestry, sources, and directions of gene flow.

### 4.1 Demography Reconstruction of the European Crows With Neutral Loci

Western European crows share the all‐black plumage of crows from Spain but are genomically more similar to grey‐coated hooded crows, except for a single aberrant locus on chr18 and other locally confined genomic regions (Knief et al. 2019; Poelstra et al. 2014; Vijay et al. 2016). Our demographic analysis reveals that this pattern results from high levels of gene flow homogenising the genomes of Western European carrion crows and Northern and Southern European hooded crow populations, except for the colour locus (‘genome‐wide swamping’). Interpreting parameter estimates of the best‐fit model in the context of biogeographic knowledge from other studies (Hewitt 1999, 2000, 2001; Pons et al. 2019; Taberlet et al. 1998; Taberlet and Bouvet 1997), we retrace the evolutionary history of crows in Europe as follows.

Based on parsimony within and beyond the species complex, the ancestral state was all‐black (Londei 2013; Vijay et al. 2016). The all‐black carrion crow ancestor split from the ancestor of all hooded crows 428 to 504 kya, which received a mutation on chr18 shortly after, inducing a pied phenotype. This is consistent with divergence estimates of 343 kya between Iberian and Middle Eastern populations at the ‘pigmentation locus’ on chr18. A second, structural mutation close to the gene *NDP* known to ‘embellish’ the pied phenotype (Knief et al. 2019; Poelstra et al. 2015), has been estimated to arise around 520 kya ago in the common ancestor of carrion and hooded crows to then sweep through the hooded crow populations (Weissensteiner et al. 2020).

Between 107 and 131 kya, during the Last Interglacial warm period, hooded crows diversified and dispersed from their initial Middle Eastern refugium to the Balkans, which represent the closest ancestral source for contemporary hooded crows across North, South, and East Europe. While the Italian Peninsula is a potential refugium for multiple European species (Brito 2005; Pellegrino et al. 2014), the genetic differentiation between the Balkan and Italian populations (*F*_ST_ = 0.0095) is comparable to that of Northern and Eastern European crow populations (*F*_ST_ = 0.007 to 0.0124). If a refugial population existed in the Italian Peninsula, it likely went extinct during the Pleistocene and has not contributed measurably to the ancestry of present‐day hooded crows. Instead, the homogeneity of the contemporary hooded crow population supports expansion from a single refugium in the Middle East, with a possible secondary refugium in the Balkans during the last ice age.

In contrast, the carrion crow remained restricted to the Iberian refugium throughout most of the Würm glaciation, likely due to the extensive ice sheets across the Pyrenées, which acted as a strong natural barrier (Stewart et al. 2010). Around 31.2 to 34.5 kya, the ancestors of contemporary Western European crows dispersed from the Iberian refugium and may have moved east of the Pyrenées. Although global temperatures were generally low and ice sheets were extensive during this period, a slight warming may have created a leaky refugium, providing opportunities for crows from Spain to disperse across the Pyrenées (O'Regan 2008). Additionally, an unidentified bird bone recovered from a layer in the Pyrenées dated to 35.0 to 39.0 kya supports the possibilities of avian life in the region during the Würm glaciation (Samper Carro et al. 2024). Therefore, carrion crows may have dispersed out of Iberia prior to the Last Glacial Maximum (LGM), over the Pyrenées and formed a source population for the subsequent Western European expansion during warming.

Overall, the migration routes of carrion and hooded crows are similar to the distribution patterns of brown bears, with Iberian crows spreading into Western Europe and Balkan crows extending into other parts of Europe (Taberlet and Bouvet 1997; Waits et al. 2000). As the ice sheets gradually retreated towards the end of the Pleistocene and the LGM, populations at the edges of the refugial range expanded into newly habitable areas (Hewitt 1999). Similar to brown bears and many other species reviewed by Hewitt (1999), the Pyrenees became more porous towards the end of the glacial period, as Iberian crows and several other bird species dispersed across the Pyrenees into Western and Central Europe (Brito 2005, 2007; Pons et al. 2016, 2011, 2019; Pritchard et al. 2000). In contrast, hooded crows in Italy, likely having populated from the Balkans, were unable to cross the Italian Alps, suggesting that the Alps continued to act as a strong barrier during the same period. The estimated time of secondary contact between carrion and hooded crows is between 20.0 to 30.6 kya, slightly before the end of the LGM around 19.0 to 20.0 kya. This timing predates previous estimates assuming that rapid warming opened up suitable crow habitat including breeding opportunities in larger shrubs or trees only after the intermission of a cooling phase in the younger dryas 12,800 to 11,500 years ago (Giesecke et al. 2017; Goslar et al. 2000; Rasmussen et al. 2014). Our estimate thus likely reflects either an overestimation of the time of secondary contact (intrinsically linked to an underestimation of gene flow rate) or the availability of suitable habitats for crows across Europe, the latter supported by the presence of various avian species dated to the late Pleistocene across Europe (Núñez‐Lahuerta et al. 2018). A more precise estimate of the time of secondary contact and rate of gene flow could be achieved through ancient DNA from the late Pleistocene, which would provide direct insights into the past.

Aside from the Italian Alps, the hybrid zone between carrion and hooded crows also extends along the river Elbe, one of Europe's major rivers. The Elbe flows through the northern part of the Czech Republic, traverses Germany, and empties into the North Sea. As a natural geographic barrier, the river can limit dispersal and lead to the formation of different habitats on each side, contributing to the formation of a hybrid zone. This hybrid zone is shared by a variety of organismic groups, including the chalkhill blue butterfly (*Polyommatus coridon*) (Schmitt and Zimmermann 2012), the fire‐bellied toad ( *Bombina bombina* ) (Szymura et al. 2000; Szymura and Barton 1986), and the house mouse ( *Mus musculus* ) (Ďureje et al. 2012). While the river itself is not an insurmountable barrier to the carrion and hooded crows, it may have led to lower population densities near the river, potentially stabilising a hybrid zone between two distinct populations (Barton and Hewitt 1985).

### 4.2 Genome‐Wide Swamping in a Presumably Narrow Hybrid Zone

Our study supports the observation that carrion and hooded crows on either side of the hybrid zone, including the Italian Alps and river Elbe, experience high levels of gene flow, with migration from grey‐coated hooded crows into all‐black Western European crows occurring at more than twice the rate. While the rest of the parental carrion crow ancestry of the Western European population is diluted by genomic swamping, the parental alleles encoding the all‐black phenotype are maintained by divergent selection (Knief et al. 2019).

This decoupling of phenotype encoded by few loci from the genome‐wide signature of the admixed Western European crows is attributed to strong assortative mating between the all‐black and grey‐coated crows, in which phenotypically all‐black individuals prefer to mate among themselves (Randler 2007; Rolando 1993), thus shielding the dark alleles on chr18 and the gene *NDP* from gene flow (Knief et al. 2019). However, prezygotic isolation only provides a partial barrier to gene flow, and assortative mating without reduced hybrid fitness is insufficient in keeping two diverging populations apart genome‐wide (Brodin and Haas 2009; Irwin 2020; Metzler et al. 2021).

For speciation to proceed, coupling of multiple barrier loci across the genome is necessary (Schield et al. 2024). If the diverging populations accumulated a large number of mutations across the genome serving as potential targets of selection with deleterious effects in hybrids, heterospecific ancestry would more likely prevail in regions of high recombination where it is rapidly uncoupled from alleles with deleterious effects in hybrids. In diverged taxa with genetic incompatibilities, introgression indeed appears to be elevated in regions of high recombination (Hennelly et al. 2021; Martin et al. 2019; Schumer et al. 2018). In contrast, at the early stages of divergence with no genome‐wide contribution to hybrid fitness, most of the introgressing alleles are likely to be neutral or even positively selected due to epistasis (Dagilis and Matute 2023). The latter could lead to a negative correlation between introgression and recombination rate. In the crow system, we find a slightly negative, but not significant, correlation between introgression and recombination rate. This lack of positive correlation between introgression and recombination rate is consistent with the absence of numerous barrier loci, as has similarly been observed in other recently diverging systems, such as *Drosophila melanogaster* (Dagilis and Matute 2023; Duranton and Pool 2022) and *Formica rufa* (Satokangas et al. 2024). Thus, the crow system portrays a scenario where few, large‐effect loci, subject to divergent sexual selection, resist rampant, asymmetric exchange without the additional contribution of polygenic, small‐effect barriers.

Therefore, the study reveals that rampant backcrossing and gene flow, masked by the all‐black plumage, occur well outside the morphologically stable hybrid zone and extend into Southern and Western France. These results conform to a spatially explicit forward simulation by Metzler et al. (2021), which shows that frequency‐dependent sexual selection mediated by imprinted mating preferences can maintain a steep cline in the morphological hybrid zone, while the rest of the genome homogenises over large geographic distances.

### 4.3 Pitfalls of Ancestry Inference With Gene Trees and Sampling Gap

Sampling representation of the refugial population has a profound effect in identifying the ancestry of the Western European crows. In the absence of the Iraqi crow population, the Western European crows were erroneously inferred to share closer ancestry with the hooded crow than with the carrion crow. This discrepancy is attributed to most variant sites reflecting a signal of incomplete lineage sorting and introgression, and only a few barrier sites reflecting the true ancestry. In contrast, the Iraqi population represents descendants of a refugial population with limited gene flow with the Western European crows, thus enabling the ancestry of the Western European population to be disentangled from the large number of loci with an introgression signal. The study thus serves as an extreme test case for the impact of sampling on the accurate inference of evolutionary history (Shringarpure and Xing 2014; Toyama et al. 2020). It also highlights the value of explicit demographic reconstruction, its integration with gene tree‐based methods (Nielsen and Beaumont 2009) and spatially explicit interpretation in a biogeographic framework. This multifaceted approach enabled us to disentangle the ancestry of the highly admixed Western European crow population from the conflicting signals of genome‐wide swamping. We ascertained that the single aberrant locus of the Western European carrion crows is maintained by divergent selection and remains as the last safeguard of their Iberian ancestry. Against the presumption of a stable hybrid zone, rampant backcrossing and introgression occur outside of the hybrid zone under the disguise of the all‐black plumage colour.

## Author Contributions

C.Y.G. processed the raw data, performed all analyses except demographic inference with Jaatha, and wrote the initial draft of the manuscript. D.M. performed demographic inference with Jaatha and assisted with the results and discussion of the manuscript. J.F. assisted with sample provision and coordination. J.B.W.W. devised the research idea and supervised the overall study. All authors participated in the writing of the final manuscript.

## Conflicts of Interest

The authors declare no conflicts of interest.

## Supporting information


Data S1

## Data Availability

Raw fastq reads newly generated in this study are deposited on NCBI (accession ID: PRJNA837738). Codes and the full bioinformatic pipeline are publicly available on GitHub at https://github.com/EvoBioWolf/2024_CORVID_demography. This research was conducted in compliance with the Nagoya Protocol.
