# Supplementary material for: Reconciling Gene Tree Discordance and Biogeography in European Crows
Source: Mol Ecol. 2025 Apr 10;34(10):e17764. doi: 10.1111/mec.17764 (PMC12051742; doi:10.1111/mec.17764)
Supplement: Supplementary file 1 — Data S1 [file MEC-34-e17764-s001.pdf]

MOLECULAR ECOLOGY

Supplemental Information for:

Reconciling gene tree discordance and biogeography in European crows

Chyi Yin Gwee, Dirk Metzler, Jérôme Fuchs, Jochen B. W. Wolf

Table of Contents:

Supplementary Text............3
Variant calling............3
Effective population sizes through time............4
Demographic inference with fastsimcoal and Jaatha............4
Supplementary Figures............8
Figure S1. Specimen of *C. (c.) capellanus* from Iraq............8
Figure S2. Number of single nucleotide polymorphisms............9
Figure S3. Summary of demographic inference methods............10
Figure S4. Population structure of European crows............11
Figure S5. Admixture graph............12
Figure S6. Unsupervised ADMIXTURE analysis............13
Figure S7. F-branch ratios............14
Figure S8. Effective population size inferred by different approaches............15
Figure S9. Pairwise comparisons of genomic differentiation............16
Figure S10. Boxplot representation of genomic divergence............17
Figure S11. Comparison of observed and expected joint site frequency spectra............18
Figure S12. Comparison of observed and expected joint-site frequency spectra............19
Figure S13. Comparison of observed and expected joint-site frequency spectra............20
Figure S14. Assessment of parametric bootstrap............21
Figure S15. Subtree topology by Twisst of four ingroup populations............22
Figure S16. Subtree topology by Twisst of three ingroup populations............23
Figure S17. Subtrees generated by iterative sampling of three populations from simulated demographic parameters............24
Figure S18. Subtrees generated by iterative sampling of four populations from simulated demographic parameters............25
Figure S19. Relationship between introgression and recombination rate............26

Supplementary Tables 27
Table S1. Summary of sample information 27
Table S2. Summary of the mean genetic diversity and Tajima's D 33
Table S3. Summary of the range of values specified for the estimation of each parameter 34
Table S4. Matrix of the mean genetic differentiation (F_{ST}) 36
Table S5. Original and bootstrap corrected (Bscor) Jaatha's estimates 37
Table S6. Original and bootstrap corrected (Bscor) fastsimcoal's estimates 38
Table S7. A list of genes found in the underestimated regions 39
Table S8. Time of divergence 40
References 41

Supplementary Text

Variant calling

Variant calling was conducted using three callers: ANGSD 0.933 (Korneliussen et al., 2014); BCFtools 1.10.2 (Danecek et al., 2021); and GATK 4.2.6.1 (McKenna et al., 2010). The variant calling parameters applied on ANGSD were as follows: "-GL 2 -doMaf 2 -doMajorMinor 1 -doCounts 1 -doDepth 1 -SNP_pval 1e-6 -ref ${ref} -doPlink 2 -doGeno -4 -doPost 1 -geno_minDepth 3 -setMinDepth 400 -setMaxDepth 6800 -setMinDepthInd 3 -minInd 121 -minMapQ 30 -minQ 30". The filter applied for variant calling with BCFtools mpileup sets a threshold of 30 for minimum mapping quality (-q) and minimum base quality (-Q). HaplotypeCaller of GATK was used to generate both variant and invariant sites (-output-mode EMIT_ALL_CONFIDENT_SITES) and retain sites with at least a median base quality (-mbq) of 30. The invariant sites retained at GATK's HaplotypeCaller before filtering out was used to generate summary statistics, such as d_{XY} and site frequency spectrum, as well as to determine monomorphic count for demographic simulations.

Overall, we found that 82.1% of the variants were identified by all three callers (**Figure S2**). The overlapping SNPs were further processed with BCFtools to remove variants with a mapping quality (MQ) of less than 40 and a QUAL score of less than 30, which implies a probability of 0.01% that the site is not a variant. Filtering was also conducted at the genotype level to mask a genotype as missing if the read depth (DP) of the individual at that site was less than 3. Finally, any sites with more than 10% missing data (F_MISSING) across individuals were removed. A total of 14,935,463 single nucleotide polymorphisms (SNPs) remained after low-quality variants, indels and repeated regions were removed. The one-dimensional site frequency spectrum (SFS) of each population was examined and an excess of variants at frequency 0.5 was observed, suggesting the presence of paralogs. Variant sites that are not on chromosome 18 and significantly differed from Hardy-Weinberg equilibrium (p-value < 0.05) were identified with VCFtools 0.1.14 (Danecek et al., 2011) and omitted, thus removing the potential paralogs. A final set of 14,822,221 biallelic SNPs was retained for downstream analysis.

Effective population sizes through time

We applied the recommended MSMC pipeline to first generate a BED file for each scaffold for the positions of uniquely mapping regions. For each whole genome individual, *bamCaller.py* was then applied to generate a masked VCF and BED file containing sites above the mean average depth for each scaffold. These three files were required to identify heterozygous blocks using *generate_multihetsep.py* for each scaffold. Default settings were used to plot the effective population size through time of each individual across all scaffolds using 19 segments (-p 1*2+15*1+1*2). A genome of at least 15x coverage was selected to represent each population for the MSMC analysis. Only the largest 100 scaffolds from putatively neutral macrochromosomes (i.e. chr1A, and chr1-5) were considered for both the MSMC2 and SMC++ analysis.

The SMC++ 1.15.2 Docker was pulled and run on the container Singularity. VCF containing unlinked and putatively neutral SNPs of each population was converted to the SMC++ input file with the option 'vcf2smc'. Effective population size of each population was inferred with the option 'estimate' with the mutation rate and a default setting of unknown ancestral allele (--polarization-error=0.5).

For Stairway Plot analysis, we used unlinked and putatively neutral SNPs from 4-fold degenerate sites, intronic and intergenic regions to obtain the folded SFS of each population with the easySFS python script (Gutenkunst et al., 2009). The two epoch blueprint files provided by the package were updated with the SFS and sample size information of each population. The length of genome was scaled to 329,623,861 bp to account for unlinked and neutral regions using the following formula: number of SNPs used / total neutral SNPs * (total neutral SNPs + total invariants neutral sites).

Demographic inference with fastsimcoal and Jaatha

Fastsimcoal 2.7 (Excoffier et al., 2021) and Jaatha 3.2.5 (https://CRAN.R-project.org/package=jaatha) (Mathew et al., 2013; Naduvilezhath et al., 2011) were used to infer whether the 'genome-wide swamping' or the 'locus-specific introgression' model is better-fitting to the observed folded joint-SFS (j-SFS) of SPA, EURw, EURns and IRQ (**see Figure S2).** A total of 11,146,221 putatively neutral SNPs was used to generate folded joint-

SFS (j-SFS) of the six population pairs with easySFS (Gutenkunst et al., 2009), with each population represented by 15 diploid individuals, except IRQ which has five diploid individuals.

fastsimcoal: The *est* and *tpl* files of each model can be accessed via our GitHub. The ranges of values specified in the *est* file for effective population size and divergence time were based on prior demographic inferences obtained by MSMC2, SMC++, and Stairway Plot. The input j-SFS files were also modified to reflect 625,134,484 monomorphic sites (column 0,0 of each j-SFS table) as scaling of parameters is conducted simultaneously by fastsimcoal.

For each of the two models, 100 independent replicates were conducted with fastsimcoal. Each replicate involved 1,000,000 coalescent simulations per cycle to estimate the expected folded j-SFS and 100 Expectation-Conditional Maximization (ECM) (Meng & Rubin, 1993) cycles to estimate the model parameters. The initial parameter values for each replicate were randomly selected from predefined ranges specified in the *est* input file. In each ECM cycle, 1,000,000 coalescent simulations were performed using the current parameter estimates to generate the expected j-SFS, which was then compared to the observed j-SFS to compute the composite likelihood. During each cycle, one parameter was optimized at a time while keeping the others fixed. This process was repeated for all 21 parameters for a total of 100 ECM cycles to maximize the composite likelihood. The replicate with the lowest difference between the observed and expected maximum likelihoods was selected as the best-fitting estimates of each model. The best-fitting estimates of each model was compared against each other by applying Akaike information criterion (AIC) (Bozdogan, 1987) to assess which is a better model. AIC was computed as $AIC = 2k - 2\ln(\log_{10}(L))$, where k is the number of parameters and $\log_{10}(L)$ is the $\log_{10}$ likelihood reported by fastsimcoal.

Parametric bootstrap was conducted on the best-fitting model by simulating 100 independent j-SFS. Each j-SFS was generated with 636,281 independent loci of 1,000 bp with a transition rate of 0.5 under a finite-site mutation model. Each simulated j-SFS consists of 636,281,000 sites, which is comparable to the total number of observed neutral variant and invariant sites (i.e. 11,146,221 and 625,134,484 respectively). The parameters for each of the 100 simulated j-SFS were re-estimated with 1,000,000 coalescent simulations and 100 ECM cycles. The initial parameter values for these estimations were set to the best-fitting model, allowing for the computation and maximization of the composite likelihood for each simulated SFS.

Parameter estimates of each bootstrap were then plotted with the model estimates to assess possible bias, given ancestral population sizes are difficult to estimate with high rates of recent gene flow between the present populations, and provide confidence intervals.

Jaatha: While Jaatha also estimates parameters by maximizing the composite likelihood of the observed and expected j-SFS, it categorizes j-SFS into fewer bins to reduce computational complexity. For each of the six population pairs, we used the folded j-SFS coarsened into 14 bins of polymorphism. In addition, Jaatha can simultaneously infer recombination rates, which could potentially improve the accuracy of demographic parameter estimations. Statistics based on violations of the four-gamete condition in pairs of polymorphic sites were applied (Hudson & Kaplan, 1985). The four-gamete condition is violated in the absence of a tree accounting for both polymorphic sites with one mutation per site. This may occur due to recombination between the two sites or double-hit mutation. As unphased data was used, we considered the condition to be violated for a pair of positions only if it was violated in every possible phasing. We calculated the fraction of pairs of sites violating the four-gamete condition in 91 putatively neutral and randomly chosen sets of 10,000 bp DNA sequence from 15 Spanish, 24 Western European, 54 north-south European, and five Iraqi crows each. The fraction of violating sites were categorized into four groups: [0, 0.1]; [0.1, 0.2]; [0.2, 0.3]; and [0.3, 1] for single population, and [0, 0.005]; [0.005, 0.01]; [0.01, 0.015]; and [0.015, 1] for population pairs. In total, 28 sets of DNA sequence fall into one of the four categories for four single populations and three population pairs. We used the R package scrm (Staab et al., 2015) to simulate 91 loci of 10,000 bp with recombination under an infinite sites model to estimate the expected values for the four-gamete condition-based statistics.

Jaatha was run with msprime (Baumdicker et al., 2022) to simulate j-SFS with 400 loci of 2,000 bp without recombination for each cycle using an infinite sites model. Scaling was done to approximate the expected SFS for full genome data, taking into account that only 800,000 bp has been simulated for a total neutral genome size of 636,250,705 (i.e. Actual neutral genome size / Simulated genome size = 7,954). Note that scaling was conducted similarly in fastsimcoal. In each iteration step of Jaatha, 1,000 simulations were performed with different combinations of parameter values. Jaatha starts with parameter combinations sampled from the entire parameter range and first applies three “zoom-in” iterations in which the range for further simulations is restricted to promising regions of the parameter space. Then, further iterations

were carried out, shifting the range of simulation parameter values around the optimum of composite likelihood. Convergence was usually detected after 30 to 50 of these iterations. This procedure was repeated five times, and for the best five parameter combinations of each of the five repetition, the composite likelihood was assessed again based on 100 new simulations. The output of Jaatha is then the parameter combination with the highest likelihood in this final step.

Parametric bootstrap was conducted by launching 100 simulations of j-SFS with the fitted parameters using both infinite and finite-sites models to assess possible bias by omitting back mutations and double hits. The simulated SFS were generated with 636,281 loci of 1,000 bp using msprime. Each simulated j-SFS consists of 636,281,000 sites, which is comparable to the total neutral genome size. For the finite-sites bootstrap simulations, we combined msprime and scrm with seq-gen (Rambaut & Grassly, 1997) and applied a HKY model with a transition rate of 0.5 (Hasegawa et al., 1985). The bootstrap simulations were repeated with fastsimcoal and vice versa to assess model fitness with a different simulator. Re-estimations of the model parameters were conducted with the same procedure as described above. Bootstrap bias correction (Efron & Tibshirani, 1994) was conducted and quantiles were calculated with default settings of the quantile function in R.

Supplementary Figures

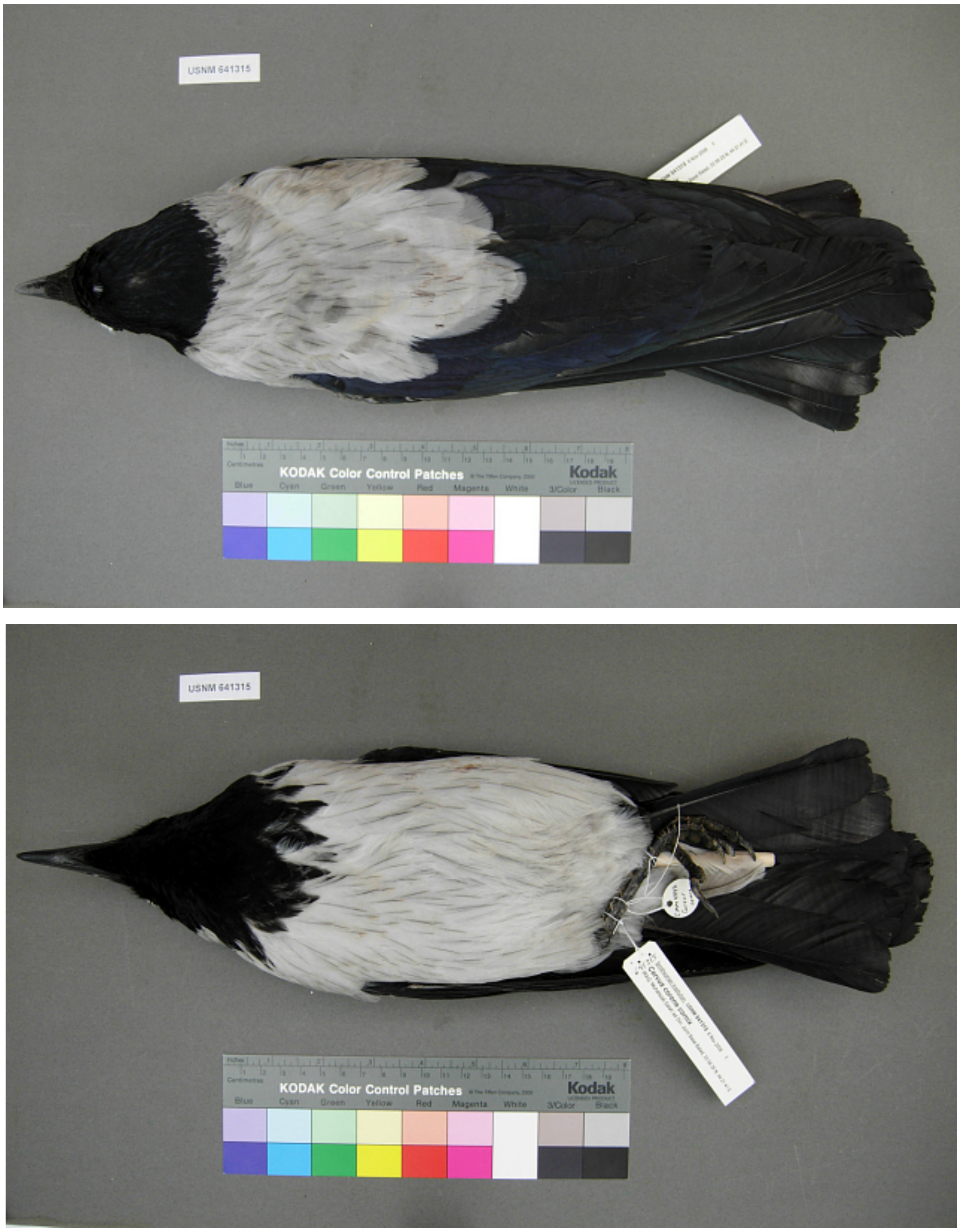

Figure S1. Specimen of *C. (c.) capellanus* from Iraq. The dorsal (top) and ventral (bottom) view of specimen USNM 641315 deposited at Smithsonian National Museum of Natural History Museum (USNM). The gray coat of *C. (c.) capellanus* is the palest among all subspecies of the hooded crow, appearing almost white. Specimen photos were created by Kevin Kerr and provided by USNM. Information about this specimen can be accessed via the museum depository: http://n2t.net/ark:/65665/385720b51-912b-459c-b8cb-6d7f4d493b27

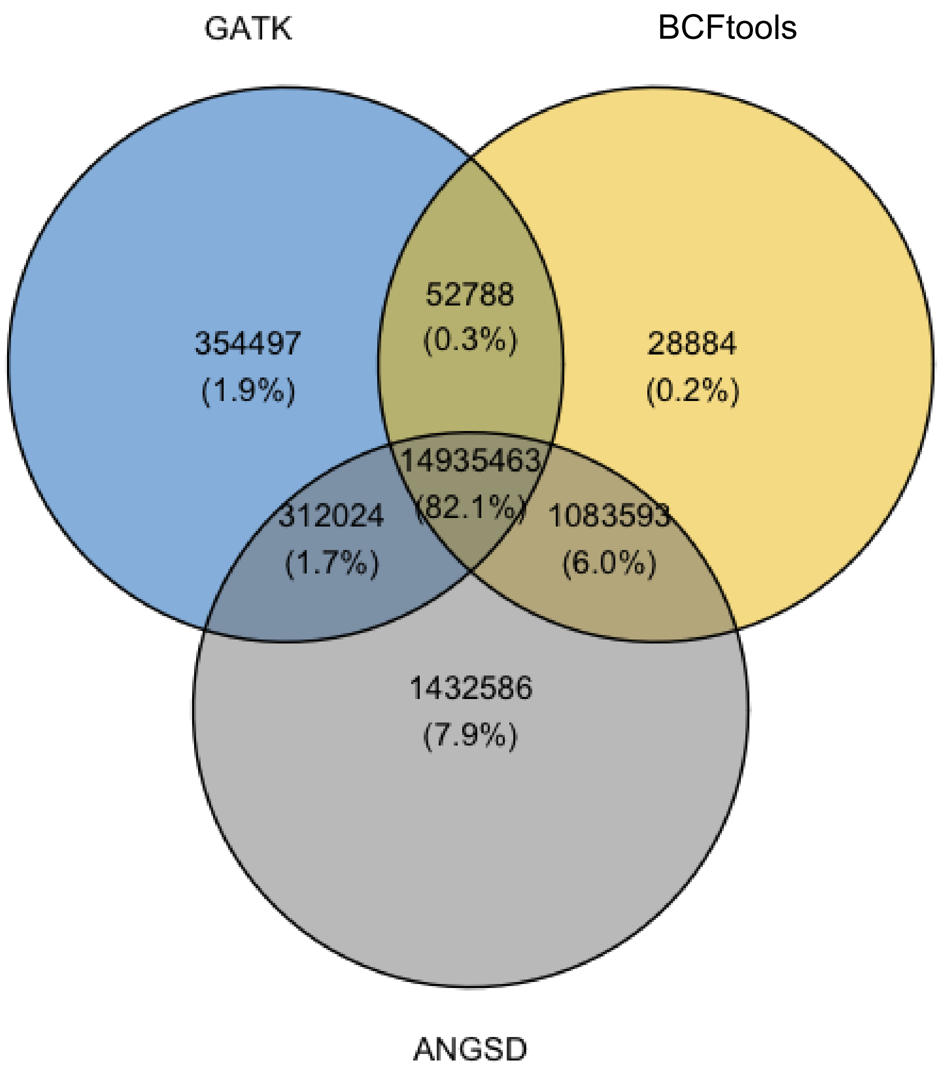

Figure S2. Number of single nucleotide polymorphisms (SNPs) among 134 individuals of the *C. coro*ne species complex discovered by each of the three callers: GATK 4.2.6.1, BCFtools1.10.2 and ANGSD 0.933. Overall, 82.1% of all possible SNPs were discovered by all three variant callers.

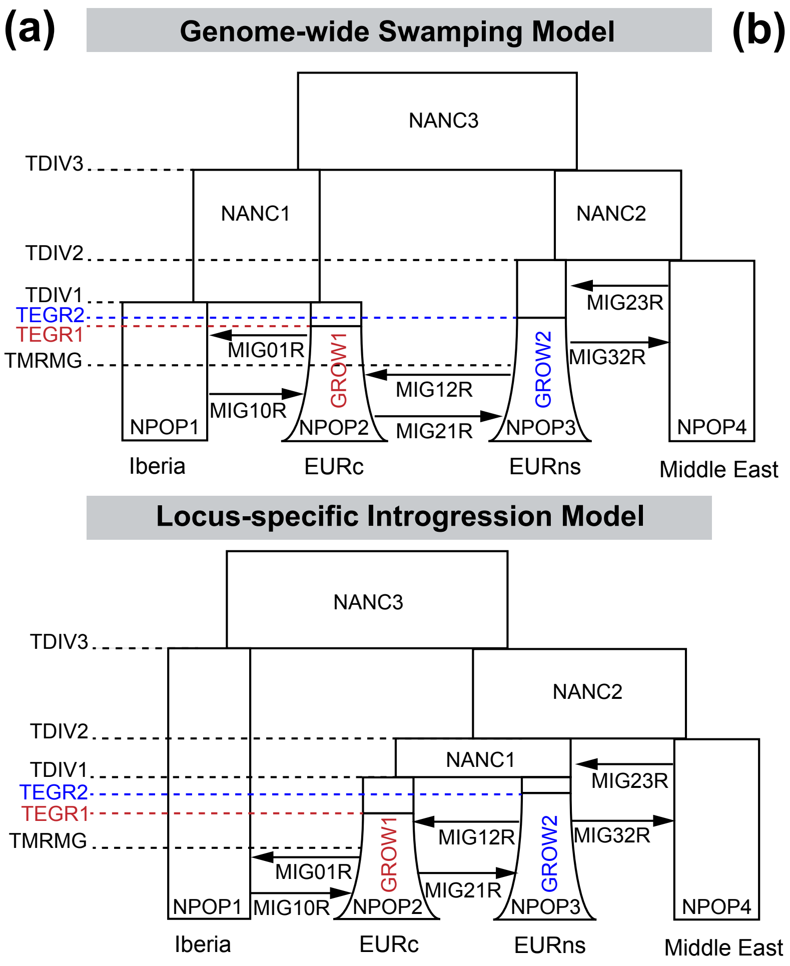

(b)

Step	fastsimcoal2	Jaatha
1	Parameter Estimation	
	- Neutral folded j-SFS<br>- Infinite sites model<br>- 100 replicates; each with 100 ECM cycles and 1M simulations per cycle with in-built Fastsimcoal simulator<br>- ECM cycle optimizes one paramter at a time	- Neutral folded j-SFS & LD stats<br>- Infinite sites model<br>- 1000 simulations per iteration with msprime & scrm<br>- 3 zoom-in iterations to search promising parameter space, then further iterations around the optimum of composite likelihoods
2	Model Comparison	
	- Select best estimates of each model from the 100 replicates based on smallest delta composite likelihoods<br>- Select best-fit model based on AIC	- Best estimates of each model based on convergence of composite likelihoods after 30-50 iterations.<br>- Select best model based on AIC
3	Cross Validation	
	- Generate SFS from the estimates of the best model of Jaatha<br>- Compare j-SFS of empirical data and simulated Jaatha estimates	- Generate SFS from the estimates of the best model of Fastsimcoal2<br>- Compare j-SFS of empirical data and simulated Fastsimcoal2 estimates
4	Parametric Bootstrap	
	- Generate 100 SFS from the estimates of Fastsimcoal2's best model with finite and infinite sites models<br>- Compare j-SFS of the empirical and simulated estimates<br>- Re-estimate each of the 100 finite-sites SFS as in step 1 with initial values	- Generate 100 SFS from the estimates of Jaatha's best model with finite and infinite sites models<br>- Compare j-SFS of the empirical and simulated estimates<br>- Re-estimate each of the 100 finite-sites SFS as in step 1 with initial values
5	Bias Correction	
	Bias-corrected estimate = $2 \times \hat{\theta}_{\text{original}} - \hat{\theta}_{\text{bootstrap mean}}$<br>Calculate confidence interval from the 2.5th and 97.5th percentile of the bootstrapped estimates for the lower and upper boundary, respectively	

Figure S3. Summary of demographic inference methods. (**a**) Schematic diagram of the two demographic models tested to discern whether the 'genome-wide swamping' or 'locus-specific introgression' model fits the empirical data better. A total of 21 parameters were estimated. **(b)** Workflow of the demographic analysis assessed using fastsimcoal2 and Jaatha.

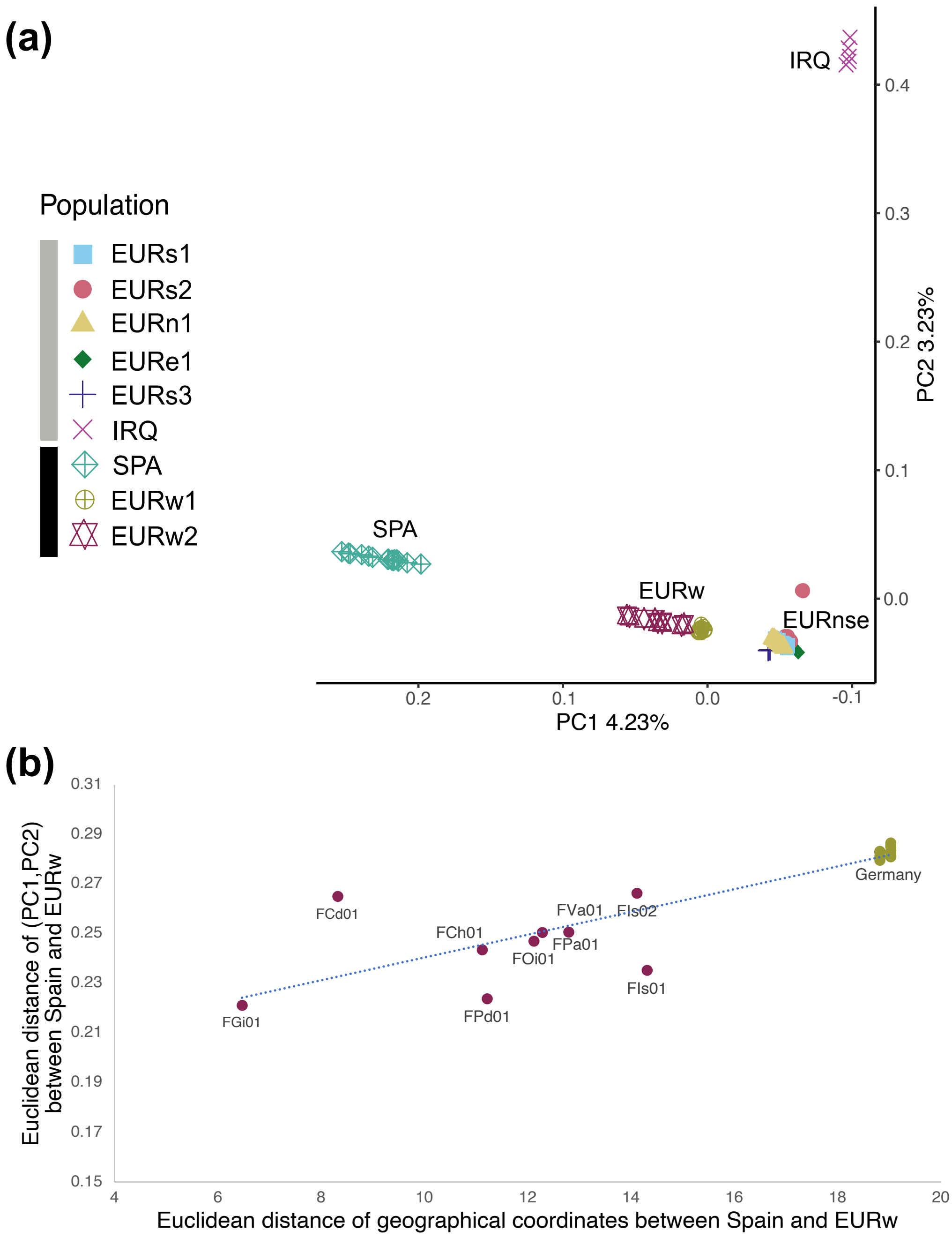

Figure S4. Population structure of European crows based on 14.8 million variants. (**a**) Principal Component Analysis shows the Spanish and Iraqi populations have the highest variation among European crows along PC1. Populations: Spain (SPA), Germany (EURw1), France (EURw2), Italy (EURs1), Bulgaria and Israel (EURs2), Poland (EURn1P), Sweden (EURn1S), Western Siberia (EURe1), Corse (EURs3), and Iraq (IRQ) (**b**) A slight clinal variation is observed among the all-black carrion crow populations (EURw2: France, EURw1: Germany), with genetic differentiation between the Spanish and EURw crows increasing as geographical distance grows.

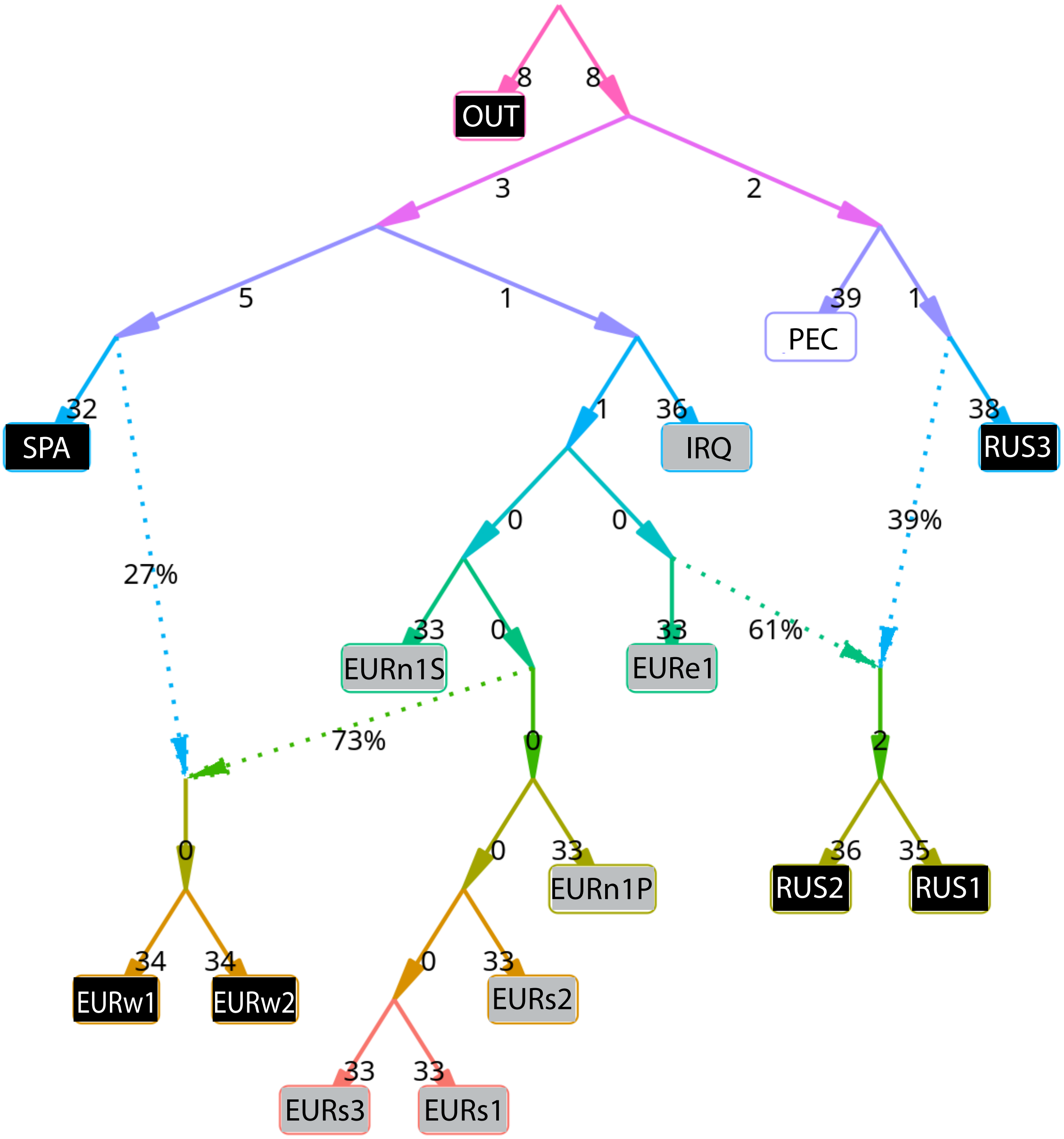

Figure S5. Admixture graph of the *C. corone* species complex with two admixture events inferred by ADMIXTOOLS2. The admixture graphs generated with a closely-related outgroup, *C. brachyrhynchos*, and a distant outgroup, *C. moneduloides*, are identical. The color of each box represents the plumage type of the population: black, gray, or pied. Populations: Spain (SPA), Germany (EURw1), France (EURw2), Italy (EURs1), Bulgaria and Israel (EURs2), Poland (EURn1P), Sweden (EURn1S), Western Siberia (EURe1), Corse (EURs3), Iraq (IRQ), central Siberia (RUS), eastern Siberia (RUS2), southeastern Siberia (RUS3), and central Asia (PEC).

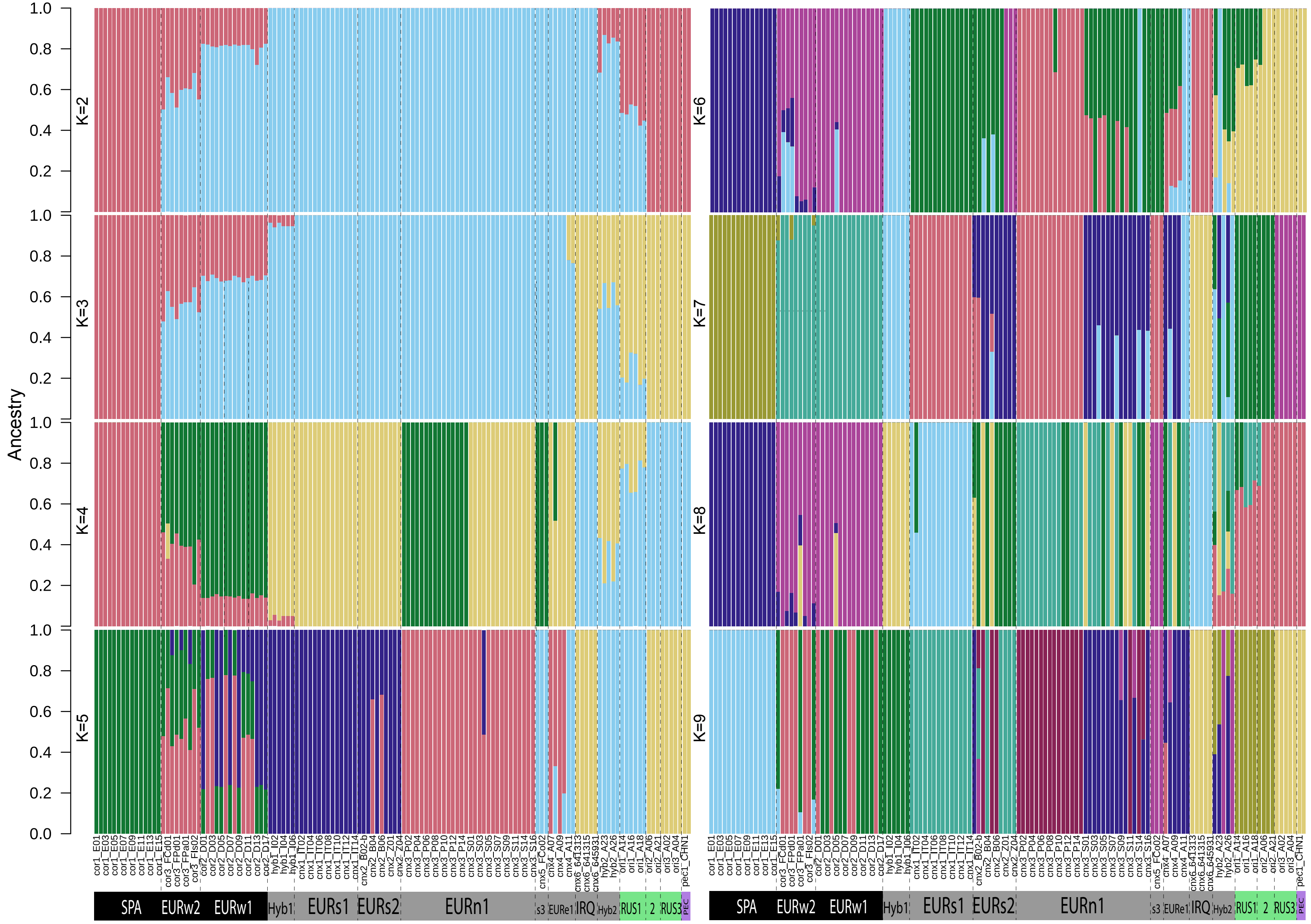

Figure S6. Unsupervised ADMIXTURE analysis of 7.7M unlinked SNPs for K=2 to 9, where K is the number of clusters.

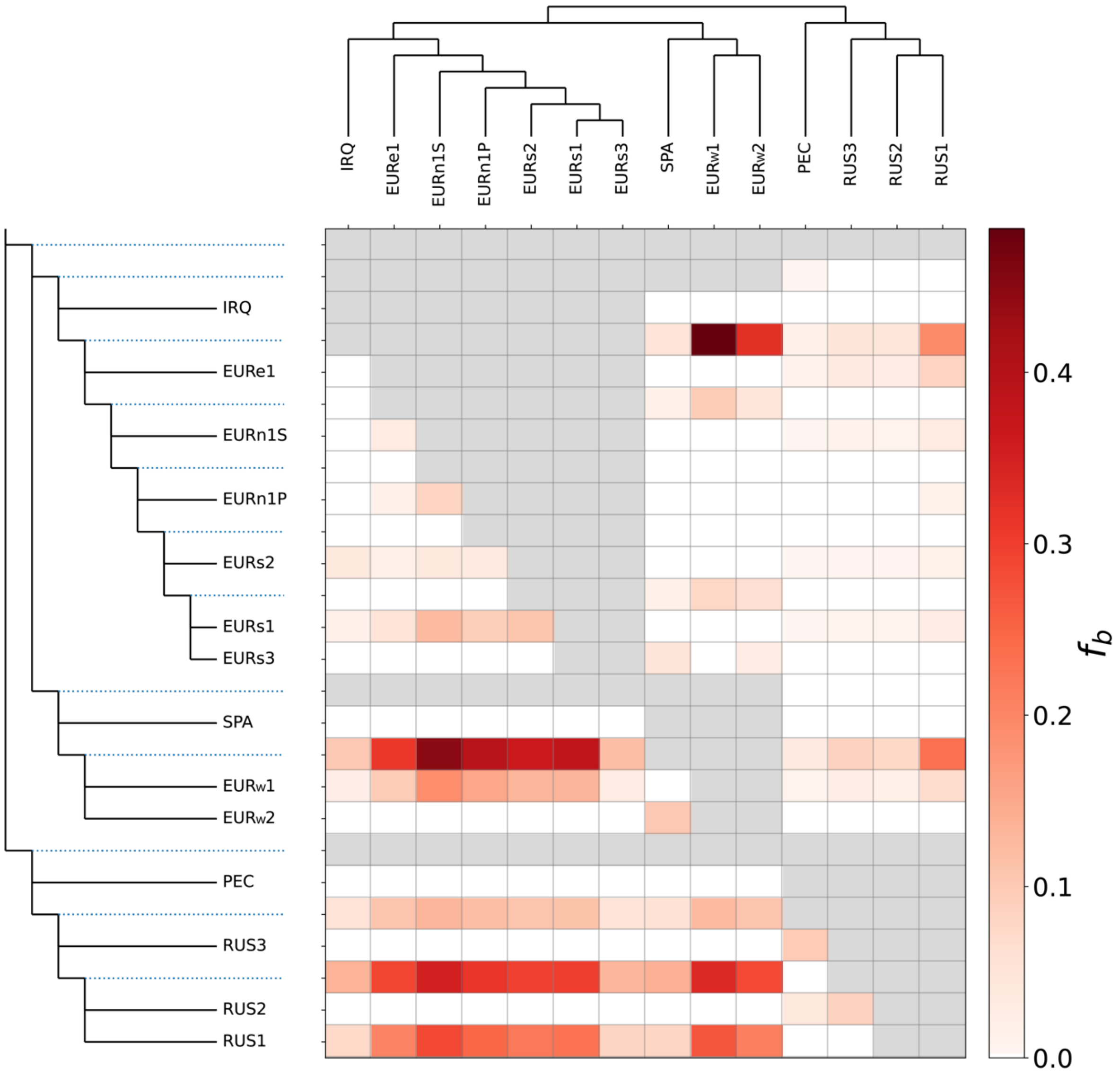

Figure S7. F-branch ratios of a predefined tree topology of the *C. corone* species complex conducted by Dsuite. Higher levels of allele sharing were detected between all-black EURw and gray-coated hooded crow populations (EURnse) than between EURw and the Iraqi population (IRQ). Similarly, higher levels of allele sharing were found between gray-coated hooded crows and EURw than with the all-black population from Spain (SPA). Populations: Spain (SPA), Germany (EURw1), France (EURw2), Italy (EURs1), Bulgaria and Israel (EURs2), Poland (EURn1P), Sweden (EURn1S), Western Siberia (EURe1), Corse (EURs3), Iraq (IRQ), central Siberia (RUS), eastern Siberia (RUS2), southeastern Siberia (RUS3), and central Asia (PEC).

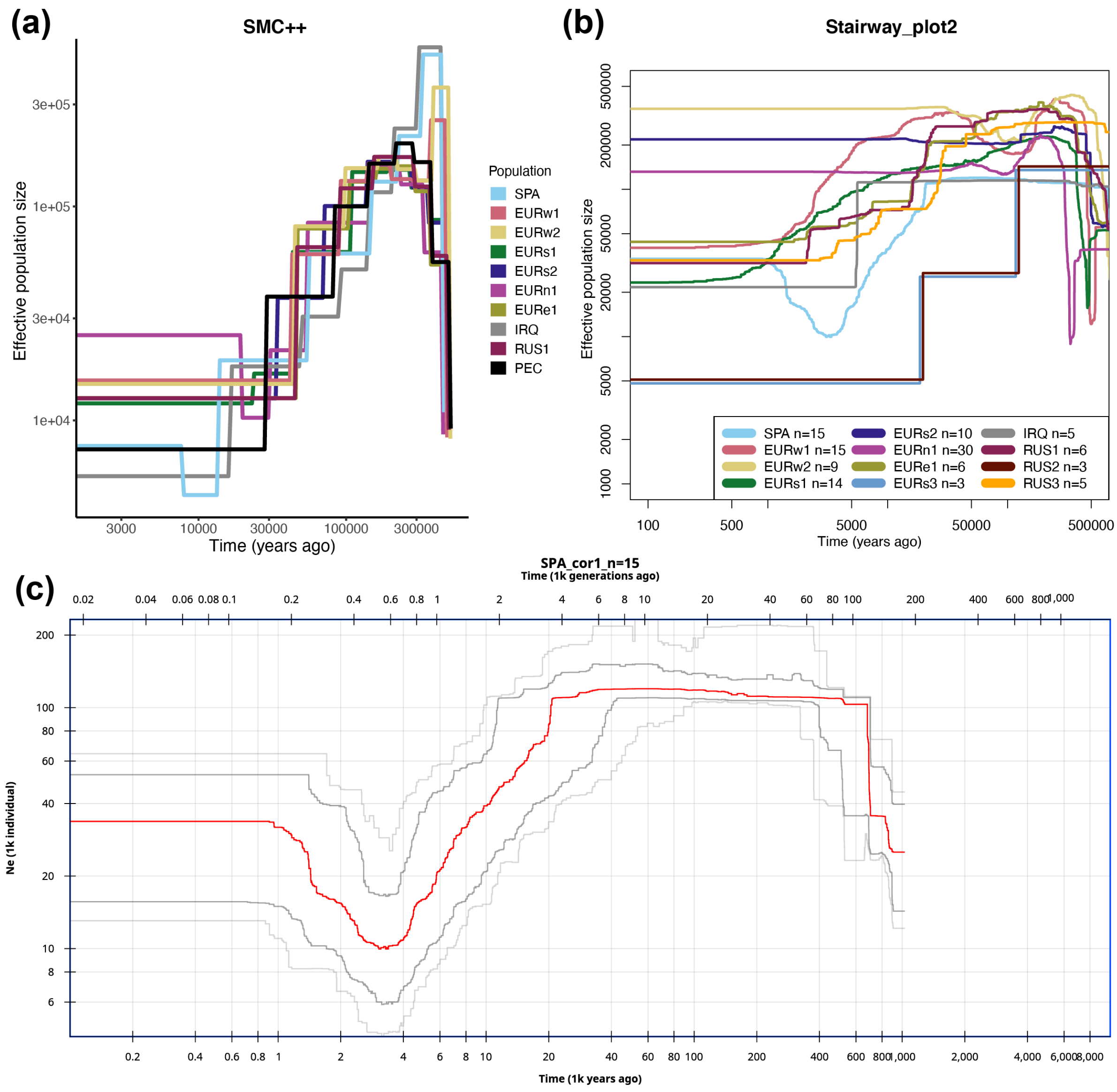

Figure S8. Effective population size inferred by different approaches. (**a**) Effective population size estimated by SMC++. (**b**) Effective population size estimated by Stairway plot2 with the respective sample size shown in the legend. Note that low sample size (n<10) could lead to poor resolution of effective size estimation in site-frequency-spectrum approach. (**c**) Stairway plot2 analysis of the Spanish population with confidence interval range shown.

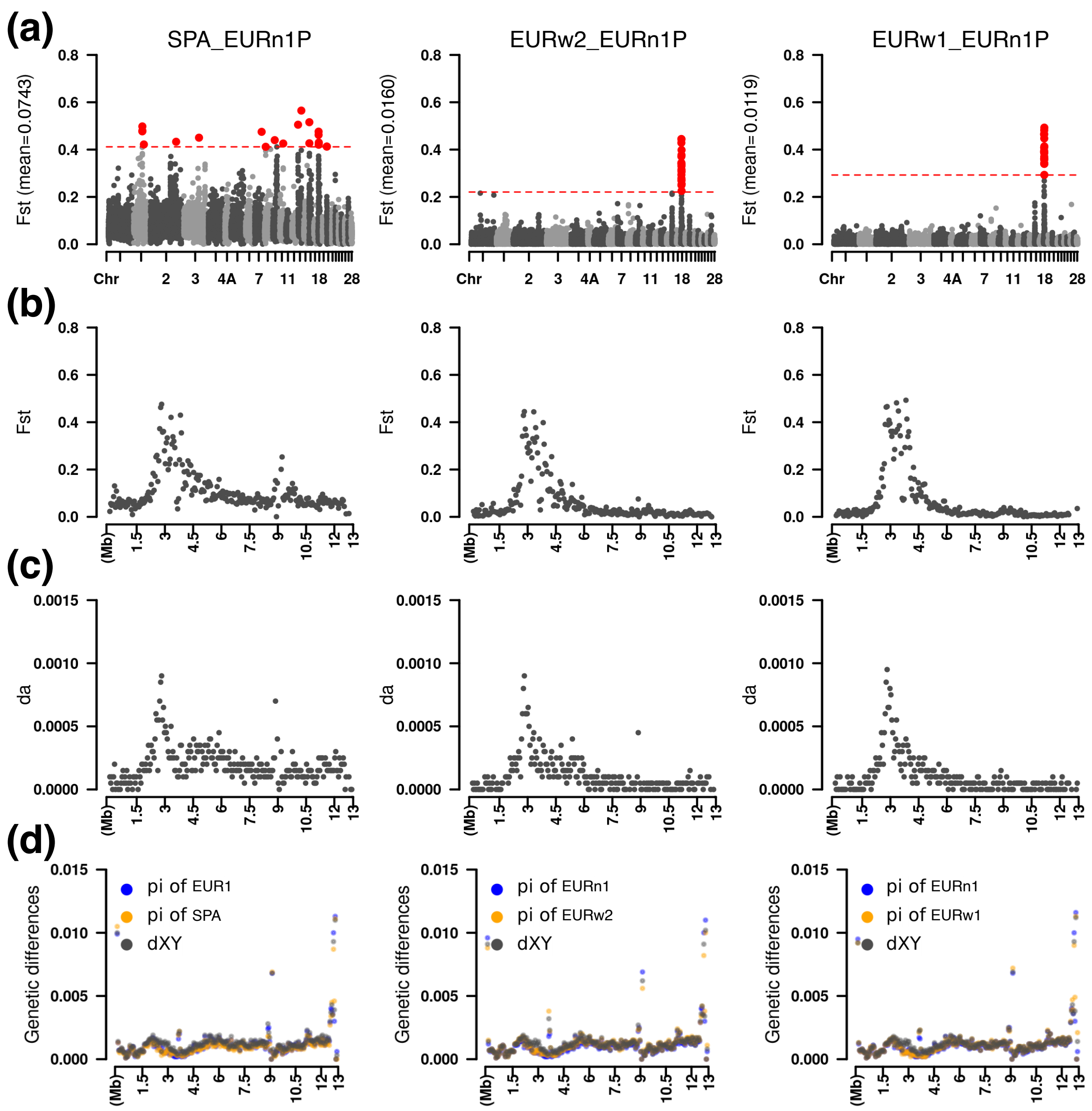

Figure S9. Pairwise comparisons of genomic differentiation between each population of all-black crows (SPA: Spain, EURw2: France, and EURw1: Germany) and gray-coated crows from Poland (EURn1P) across the genome and specifically on chromosome 18. (**a**) Mean F_{ST} of each pairwise comparison shows declining genomic divergence as the distance between all-black crows and the hybrid zone decreases, but chromosome 18 remains highly differentiated. Highly differentiated sites above the 99.9th percentile are marked in red. (**b**) The 2.5M bp peak region on chromosome 18 are in the same location and show similar F_{ST} value in all pairwise comparisons. (**c**) Net pairwise nucleotide differences (d_a) show the same pattern as F_{ST}. (**d**) Genetic differences, in the form of nucleotide diversity within each population (pi/ π) and absolute divergence between populations (d_{XY}), are reduced in the regions corresponding to elevated F_{ST}.

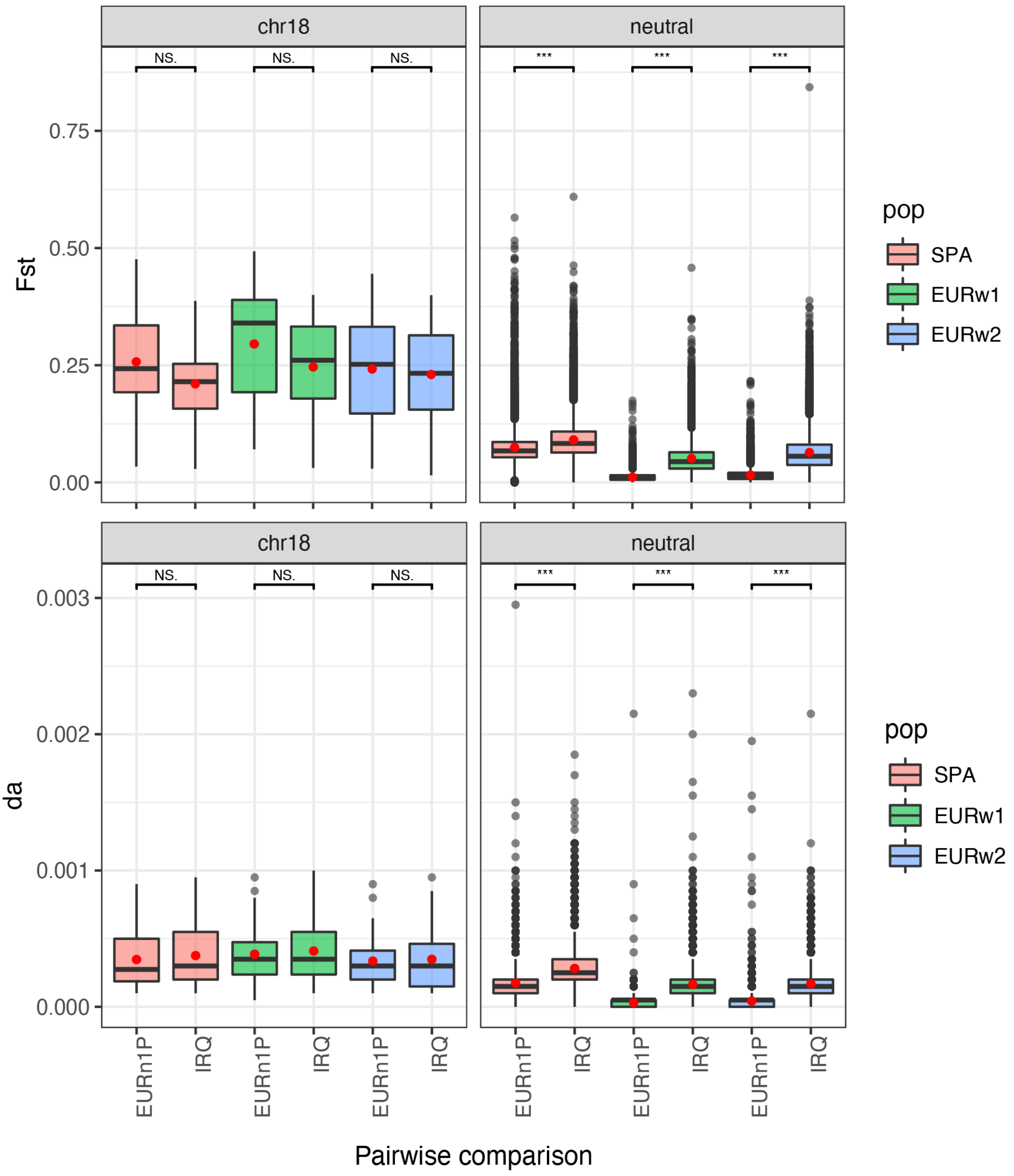

Figure S10. Boxplot representation of genomic divergence (F_{ST} and d_a) between each population of all-black crows (SPA: Spain, France: EURw2, Germany: EURw1) and gray-coated crows from either the contact zone (EURn1P: Poland) or the non-contact region (IRQ: Iraq) in the elevated region of chromosome 18 and in all other autosomal chromosome ("neutral"). The differences between each all-black crow population against EURn1P and IRQ are significant at p-value<0.001 and denoted by three asterisks (***), while insignificant comparisons are denoted by "NS."

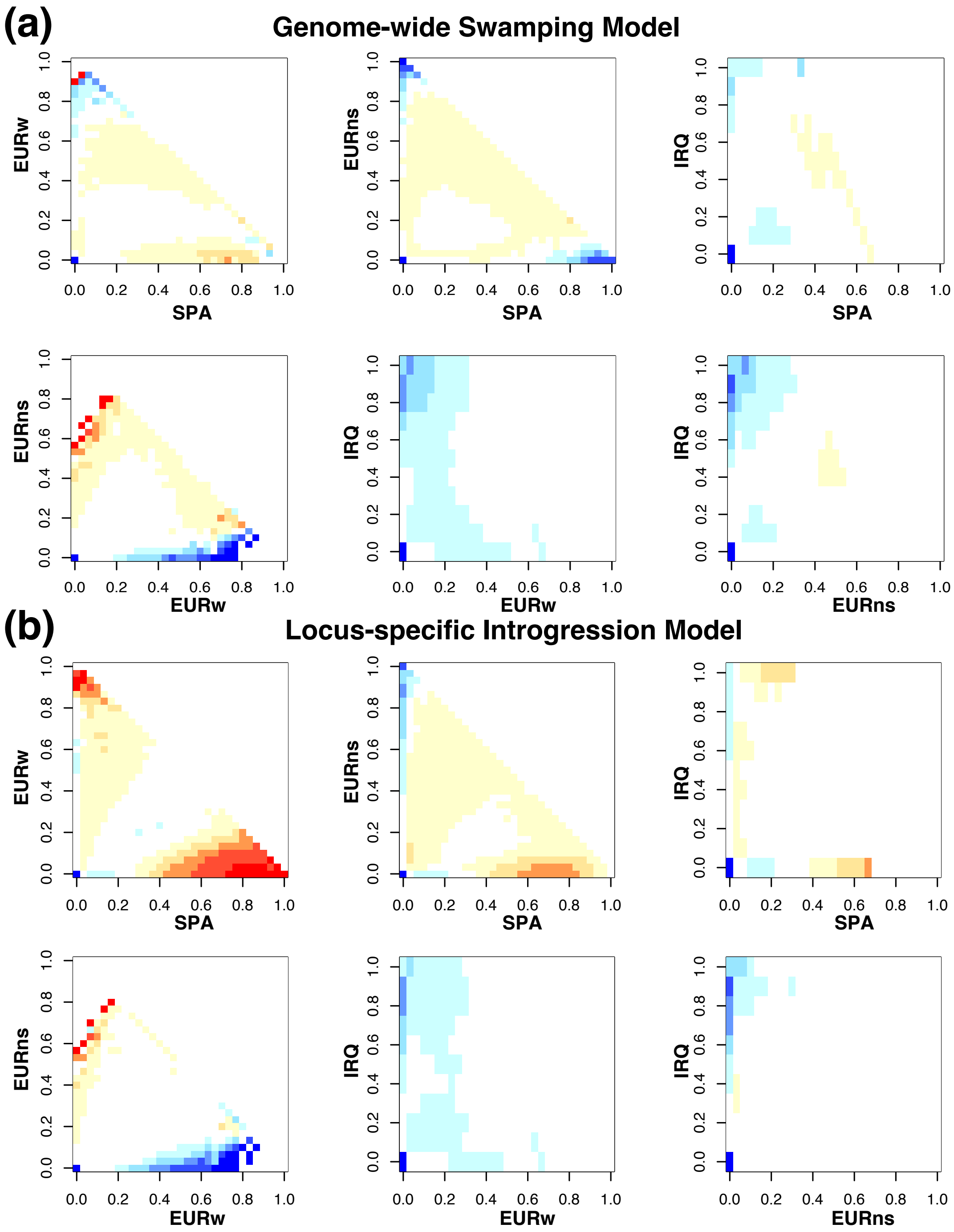

Figure S11. Comparison of observed and expected joint site frequency spectra (j-SFS), simulated with parameter estimates from Jaatha using a finite-sites model. (**a**) Expected j-SFS simulated from the parameter estimates of the best replicate of genome-wide swamping model from Jaatha. (**b**) Expected j-SFS simulated from the parameter estimates of the best replicate of locus-specific introgression model from Jaatha. Blue and red region reflects underestimated (expected < observed) and overestimated SFS classes (expected > observed), respectively.

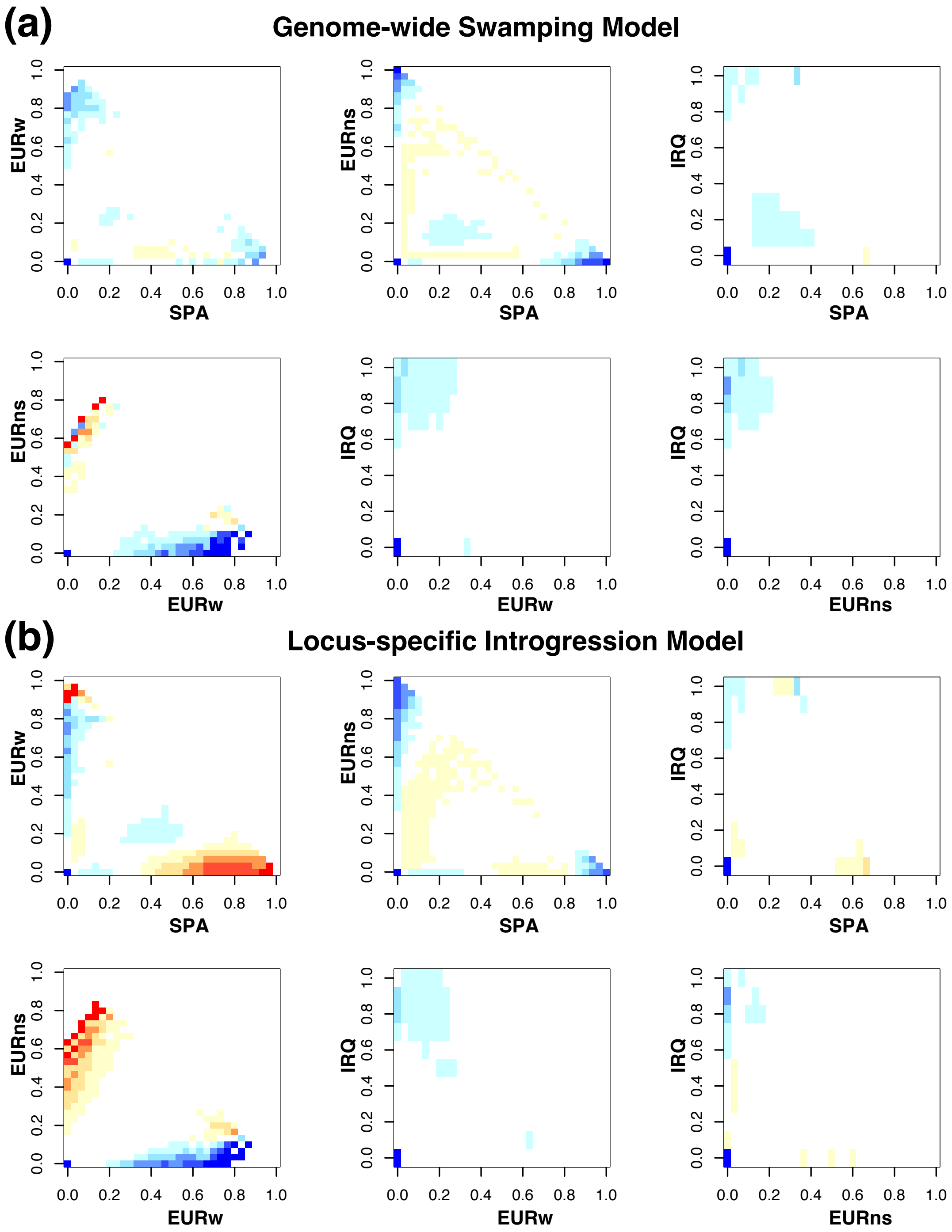

Figure S12. Comparison of observed and expected joint-site frequency spectra (j-SFS), simulated with parameter estimates from fastsimcoal using finite-sites model. (**a**) Expected j-SFS simulated from the parameter estimates of the best replicate of genome-wide swamping model from fastsimcoal. (**b**) Expected j-SFS simulated from the parameter estimates of the best replicate of locus-specific introgression model from fastsimcoal. Blue and red region reflects underestimated (expected < observed) and overestimated SFS classes (expected > observed), respectively.

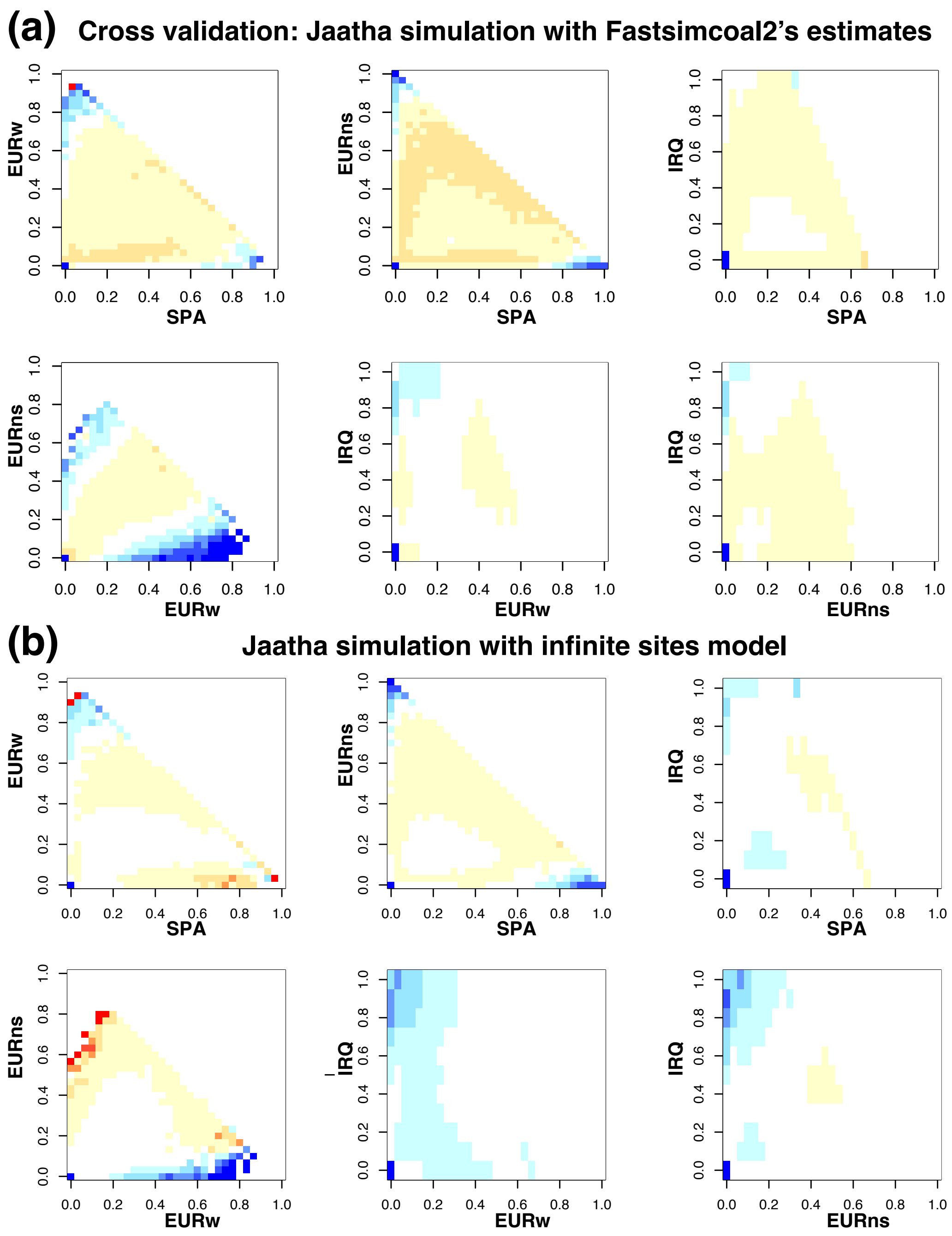

Figure S13. Comparison of observed and expected joint-site frequency spectra (j-SFS) simulated by Jaatha to cross validate the different demographic analysis approaches and mutation site-models. (**a**) Expected j-SFS simulated from the parameter estimates of the best replicate of genome-wide swamping model from fastsimcoal using finite-sites model. (**b**) Expected j-SFS simulated from the parameter estimates of the best replicate of genome-wide introgression model from Jaatha using infinite-sites model. Blue and red region reflects underestimated (expected <observed) and overestimated SFS (expected > observed), respectively.

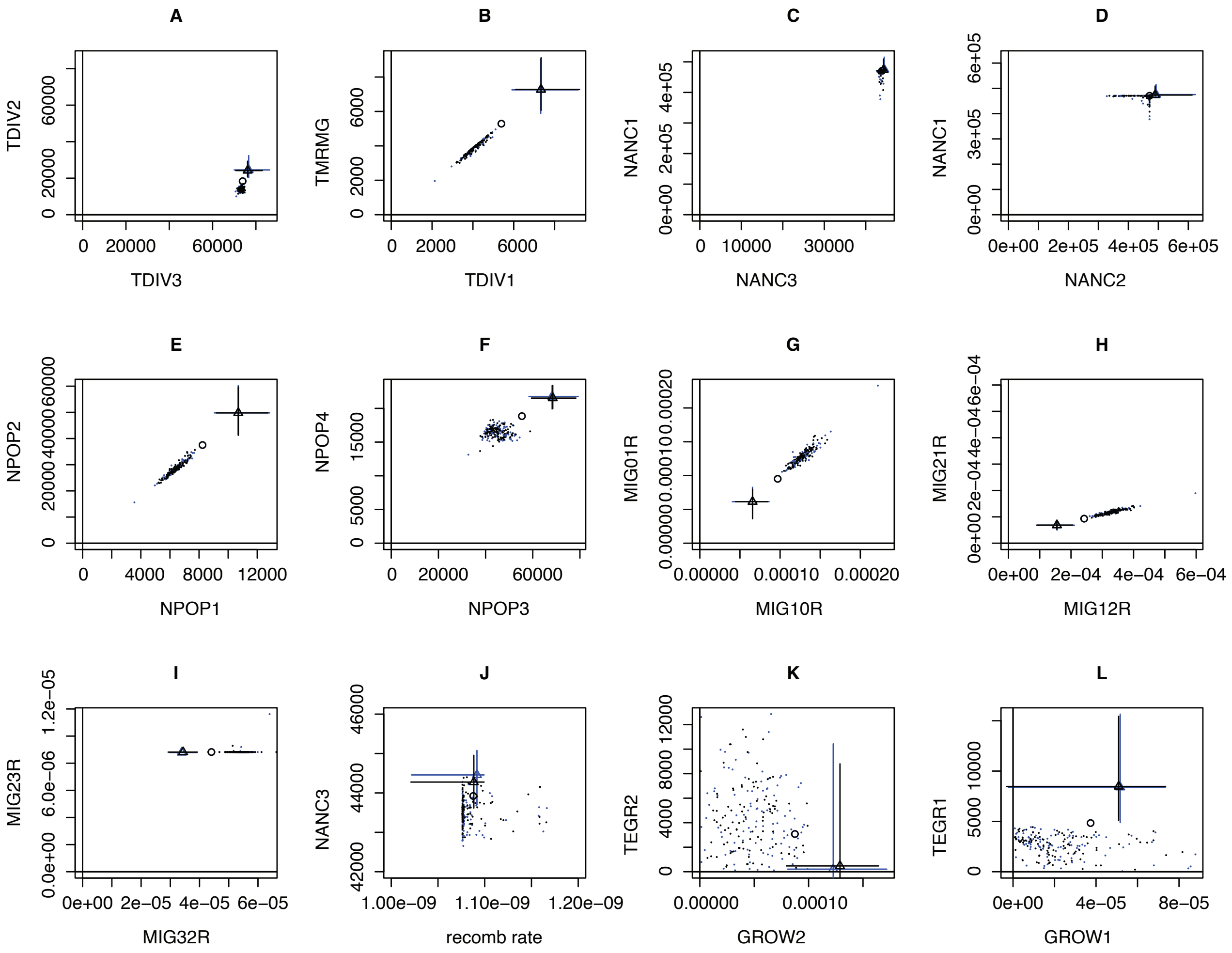

Figure S14. Assessment of parametric bootstrap for genome-wide swamping model simulated by Jaatha. Parameter estimations are denoted by open circles, estimations from parametric bootstrap simulations by tiny dots, bootstrap-based bias-corrected estimations by triangles, and confidence intervals by lines. Blue elements refers to boostrap results with finite-sites simulations and black elements to those with infinite-sites simulations.

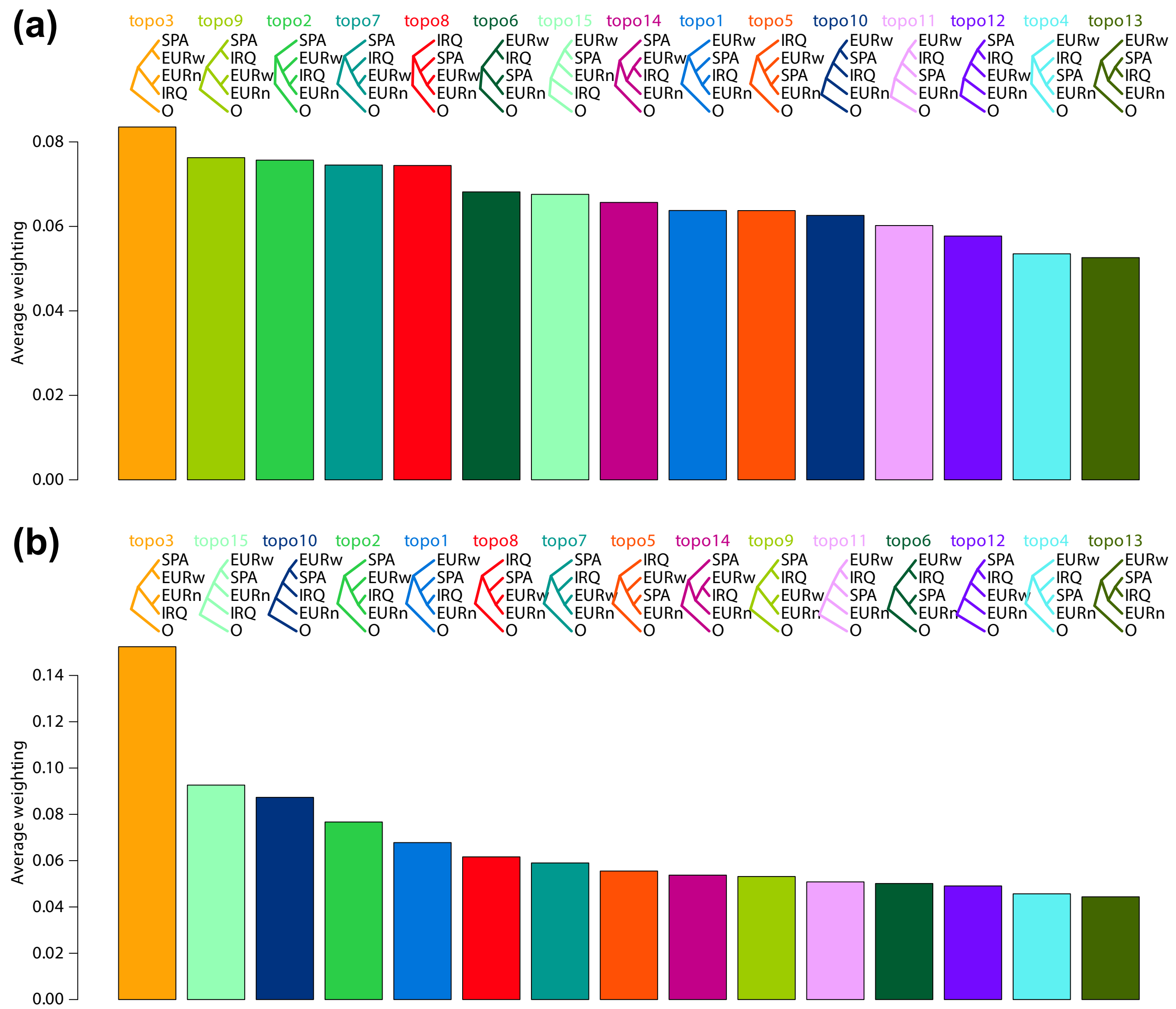

Figure S15. Subtree topology by Twisst of four ingroup populations across the genome. The inclusion of both refugial populations from Spain and Iraq correctly identifies the true ancestry of the Western European population. (**a**) Most of the SNP subtrees generated by iterative sampling of the four populations shows the correct ancestry topology even with the exclusion of the barrier sites on chromosome 18. (**b**) SNP subtrees generated from the barrier sites on chromosome 18 show high proportion of the correct ancestry topology.

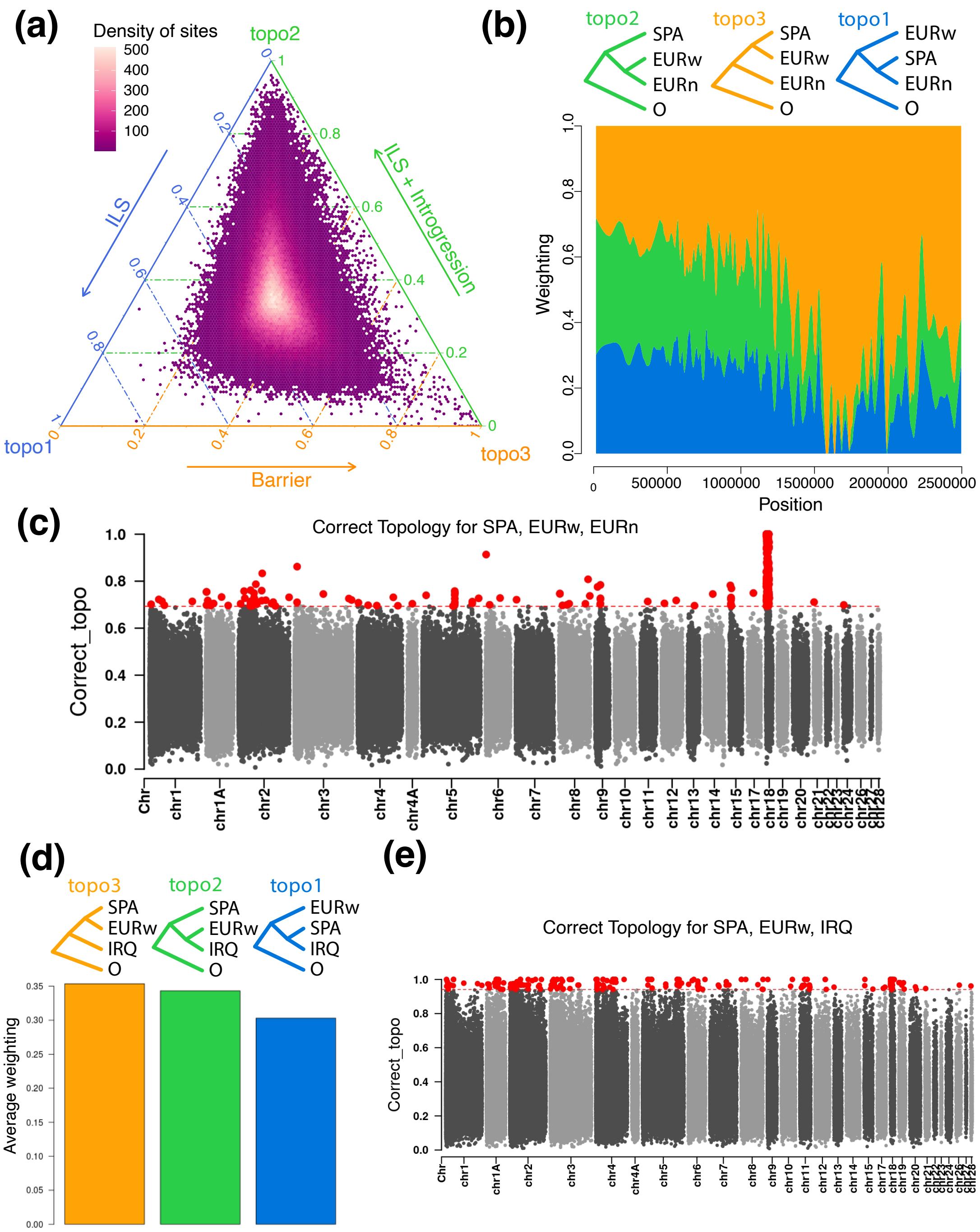

Figure S16. Subtree topology by Twisst of three ingroup populations from Spain (SPA), Germany (EURw), and Poland (EURw) (panel **a-c**) or Iraq (IRQ) (panel **d-e**). (**a**) Ternary plot of the distribution of all variant sites showing different proportion of topo1-3. (**b**) Variant sites on scaffold 78 of chromosome 18 show 100% correct topology (topo3; orange). (**c**) Weighted topology of the correct ancestry relationship across the genome. Variants above 99.9th percentile are marked in red and concentrate on chromosome 18. (**d**) Most of the SNP subtrees generated by iterative sampling of populations from Spain, Germany and Iraq shows the correct ancestry (topo3). (**e**) Weighted topology of the correct ancestry relationship across the genome. Variants above 99.9th percentile are marked in red.

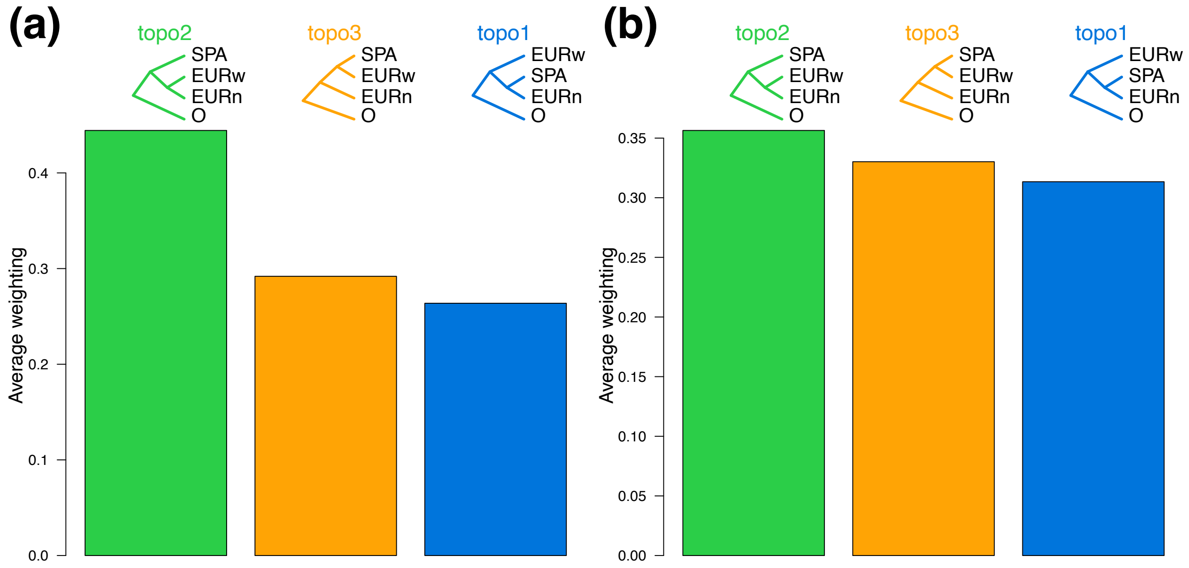

Figure S17. Subtrees generated by iterative sampling of three populations from simulated demographic parameters estimates using Fastsimcoal. (**a**) Under the 'genome-wide swamping' simulated model, most subtrees reflect an introgression signal indicating closer ancestry between EURw and EURn, instead of the true ancestry between SPA and EURw. (**b**) Under the 'locus-specific introgression' simulated model, most subtrees also reflect a closer ancestry between EURw and EURn.

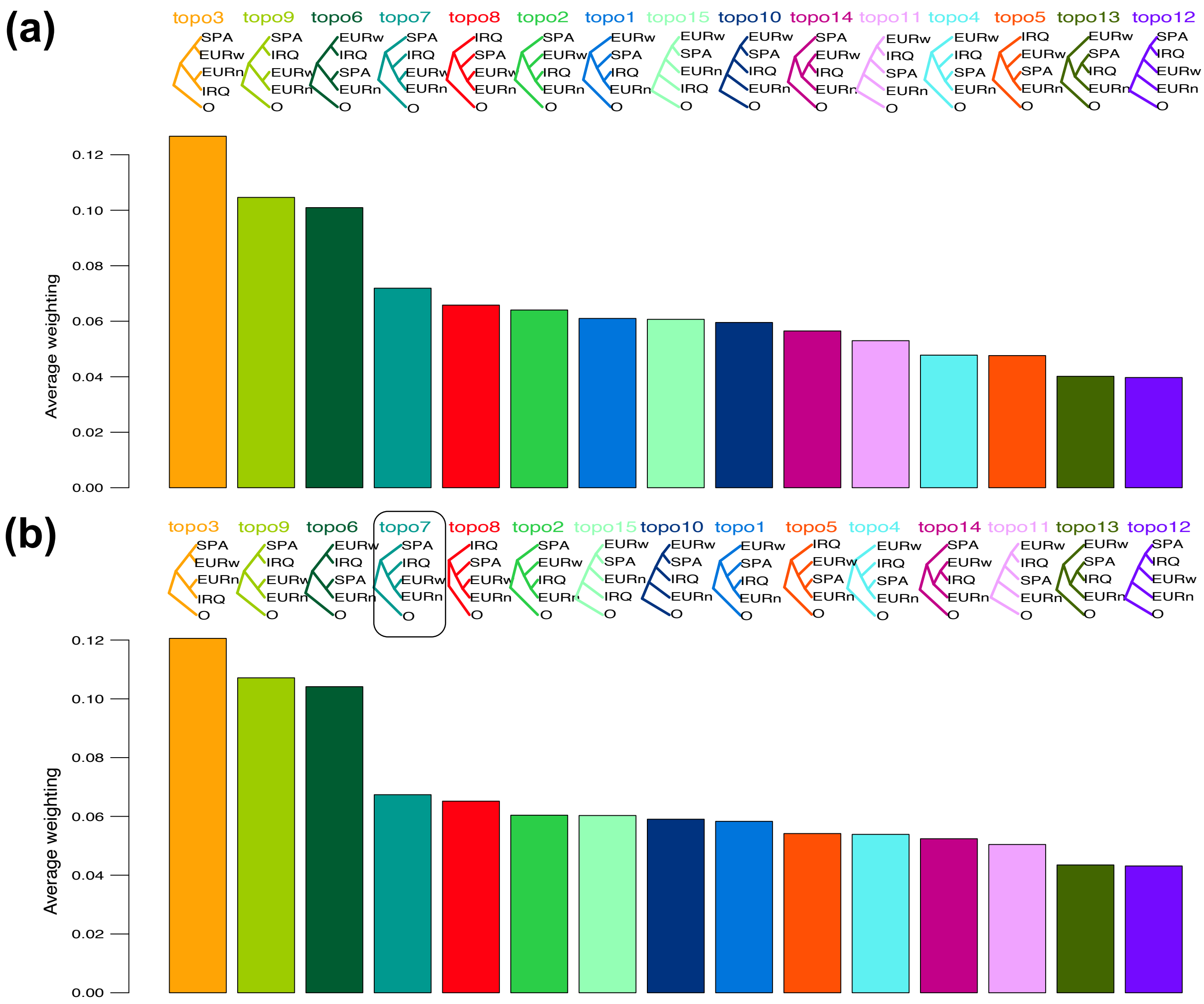

Figure S18. Subtrees generated by iterative sampling of four populations from simulated demographic parameters estimates by fastsimcoal. (**a**) Under the 'genome-wide swamping' simulated model, most subtrees reflect the correct ancestry of ((SPA,EURw),(EURn,IRQ)) as the empirical data. (**b**) Under the 'locus-specific introgression' simulated model, most subtrees also reflect a topology of ((SPA,EURw),(EURn,IRQ)), as opposed to the supposed simulated ancestry of (SPA,(IRQ,(EURn,EURw))), which is illustrated as topo7 (indicated with a black box).

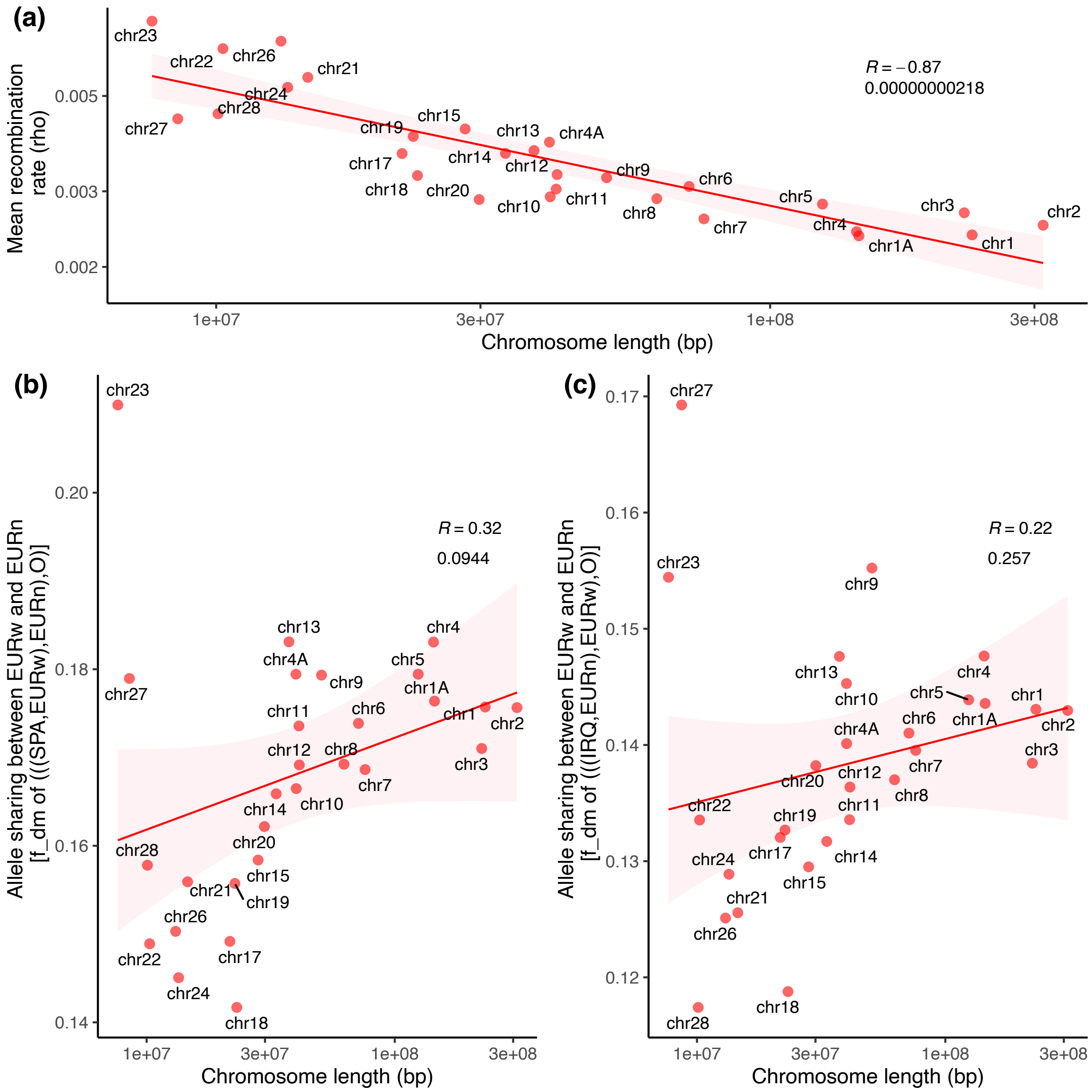

Figure S19. Relationship between introgression and recombination rate. (**a**) The expected negative correlation between mean recombination rates and chromosome length. (**b**) The degree of introgression (f_{dM}) between contact populations (EURw and EURn) for the test (((SPA,EURw),EURn),outgroup) across genomic regions of varying chromosome lengths. (**c**) The degree of introgression (f_{dM}) between contact populations (EURw and EURn) for the test (((IRQ,EURn),EURw),outgroup) across genomic regions of varying chromosome lengths.

Supplementary Tables

Table S1. Summary of sample information used in this study, including the corresponding Biosample ID on NCBI, museum voucher (if applicable), and the mean sequencing depth of each sample. The isolate ID of the 17 newly sequenced individuals are in bold. Populations: Spain (SPA), Germany (EURw1), France (EURw2), Italy (EURs1), Bulgaria and Israel (EURs2), Poland (EURn1P), Sweden (EURn1S), Western Siberia (EURe1), Corse (EURs3), Iraq (IRQ), central Siberia (RUS), eastern Siberia (RUS2), southeastern Siberia (RUS3), and central Asia (PEC).

Pop ID	Isolate ID	Museum voucher	Biosample ID	Average depth
IRQ	**IRQ_USNM_641313**	USNM 641341	SAMN28229761	24.5
IRQ	**IRQ_USNM_641314**	USNM 641313	SAMN28229762	80.4
IRQ	**IRQ_USNM_641315**	USNM 641314	SAMN28229763	20.4
IRQ	**IRQ_USNM_641341**	USNM 641315	SAMN28229760	22.0
IRQ	**IRQ_USNM_645931**	USNM 645931	SAMN28229764	18.6
EURw2	**F_Cd_01**	MNHN ZO 2016-023	SAMN28229748	16.6
EURw2	**F_Ch_01**	BOUM 2016.18.1	SAMN28229749	15.4
EURw2	**F_Gi_01**	MNHN ZO 2017-403	SAMN28229753	13.7
EURw2	**F_Is_01**	MNHN ZO 2016-022	SAMN28229754	14.3
EURw2	**F_Is_02**	MNHN ZO 2017-359	SAMN28229755	10.5
EURw2	**F_Oi_01**	MNHN ZO 2016-587	SAMN28229756	13.5
EURw2	**F_Pa_01**	MNHN ZO 2018-658	SAMN28229757	17.2
EURw2	**F_Pd_01**	MNHN ZO 2014-131	SAMN28229758	14.8

EURw2	**F_Va_01**	MNHN Uncat CDS Buoux 257-2014	SAMN28229759	15.2
EURs3	**F_Co_01**	MNHN Uncat 20-22	SAMN28229750	14.6
EURs3	**F_Co_02**	MNHN Uncat 20-23	SAMN28229751	14.1
EURs3	**F_Co_03**	MNHN Uncat 20-24	SAMN28229752	13.7
SPA	E_Vi_C01	NA	SAMN02743833	16.1
SPA	E_Vi_C05	NA	SAMN02743834	12.2
SPA	E_Vi_C08	NA	SAMN02743835	8.4
SPA	E_Vi_C14	NA	SAMN02743836	8.4
SPA	E_Vi_C19	NA	SAMN02743837	11.6
SPA	E_Vi_C22	NA	SAMN02743838	8.1
SPA	E_Vi_C23	NA	SAMN02743839	12.3
SPA	E_Vi_C32	NA	SAMN02743840	9.4
SPA	E_Vi_C37	NA	SAMN02743841	8.4
SPA	E_Vi_C44	NA	SAMN02743842	10.3
SPA	E_Vi_C46	NA	SAMN02743843	7.8
SPA	E_Vi_C48	NA	SAMN02743844	10.1
SPA	E_Vi_C51	NA	SAMN02743845	8.7
SPA	E_Vi_C57	NA	SAMN03890337	26.7
SPA	E_Vi_C58	NA	SAMN03890341	18.2
EURw1	D_Ko_C02	NA	SAMN02743818	12.1
EURw1	D_Ko_C04	NA	SAMN02997745	19.4
EURw1	D_Ko_C08	NA	SAMN02743821	7.7
EURw1	D_Ko_C11	NA	SAMN02743822	9.9
EURw1	D_Ko_C13	NA	SAMN03890325	20.2
EURw1	D_Ko_C15	NA	SAMN03890334	25.4
EURw1	D_Ko_C19	NA	SAMN02743825	14.6
EURw1	D_Ko_C20	NA	SAMN02743826	11.7
EURw1	D_Ra_C01	NA	SAMN02743827	11.0
EURw1	D_Ra_C05	NA	SAMN03890351	18.0

EURw1	D_Ra_C06	NA	SAMN03890344	7.6
EURw1	D_Ra_C14	NA	SAMN02743831	7.5
EURw1	D_Ra_C16	NA	SAMN03890329	20.6
EURw1	D_Ra_C11	NA	SAMN02743830	11.8
EURw1	D_Ko_C05	NA	SAMN02743820	10.1
EURs1	ITA_Ro_H01	NA	SAMEA3334521	12.6
EURs1	ITA_Ro_H02	NA	SAMEA3334529	9.6
EURs1	ITA_Ro_H03	NA	SAMEA3334545	9.7
EURs1	ITA_Ro_H04	NA	SAMEA3334551	8.9
EURs1	ITA_Ro_H05	NA	SAMEA3334513	6.8
EURs1	ITA_Ro_H06	NA	SAMEA3334543	12.0
EURs1	ITA_Ro_H07	NA	SAMEA3334523	13.1
EURs1	ITA_Ro_H08	NA	SAMEA3334515	7.9
EURs1	ITA_Ro_H09	NA	SAMEA3334511	9.3
EURs1	ITA_Ro_H10	NA	SAMEA3334531	7.3
EURs1	ITA_Ro_H11	NA	SAMEA3334533	13.5
EURs1	ITA_Ro_H12	NA	SAMEA3334549	15.7
EURs1	ITA_Ro_H13	NA	SAMEA3334547	12.6
EURs1	ITA_Ro_H14	NA	SAMEA3334519	10.5
EURs2	ISR_TA_H01	NA	SAMEA3334535	13.0
EURs2	ISR_TA_H02	NA	SAMEA3334539	14.8
EURs2	ISR_TA_H04	NA	SAMEA3334537	13.2
EURs2	B_So_H01	NA	SAMEA3334498	14.9
EURs2	B_So_H02	NA	SAMEA3334527	12.4
EURs2	B_So_H03	NA	SAMEA3334499	15.4
EURs2	B_So_H04	NA	SAMEA3334553	14.7
EURs2	B_SZ_H01	NA	SAMEA3334517	11.9
EURs2	B_SZ_H02	NA	SAMEA3334525	11.8
EURs2	B_un_H01	NA	SAMEA3334541	14.0
EURn1P	PL_Wa_H02	NA	SAMN02439805	8.6
EURn1P	PL_Wa_H03	NA	SAMN02439806	8.0
EURn1P	PL_Wa_H05	NA	SAMN02439807	8.2
EURn1P	PL_Wa_H06	NA	SAMN02439808	8.8
EURn1P	PL_Wa_H09	NA	SAMN02439809	11.6

EURn1P	PL_Wa_H11	NA	SAMN02439810	10.2
EURn1P	PL_Wa_H14	NA	SAMN02439811	9.6
EURn1P	PL_Wa_H16	NA	SAMN02439812	11.7
EURn1P	PL_Wa_H17	NA	SAMN02439813	7.6
EURn1P	PL_Wa_H22	NA	SAMN03890347	14.1
EURn1P	PL_Wa_H23	NA	SAMN03890353	14.1
EURn1P	PL_Wa_H52	NA	SAMN02439819	10.6
EURn1P	PL_Wa_H32	NA	SAMN02439816	11.3
EURn1P	PL_Wa_H35	NA	SAMN02439817	10.6
EURn1P	PL_Wa_H50	NA	SAMN02439818	8.3
EURn1S	S_Up_H03	NA	SAMN03890338	23.0
EURn1S	S_Up_H09	NA	SAMN03890350	25.5
EURn1S	S_Up_H16	NA	SAMN02439827	8.3
EURn1S	S_Up_H24	NA	SAMN03890346	19.0
EURn1S	S_Up_H29	NA	SAMN03890343	29.0
EURn1S	S_Up_H37	NA	SAMN03890348	10.9
EURn1S	S_Up_H43	NA	SAMN02439831	12.4
EURn1S	S_Up_H47	NA	SAMN03890345	18.3
EURn1S	S_Up_H51	NA	SAMN02439833	10.3
EURn1S	S_Up_H52	NA	SAMN02439834	10.9
EURn1S	S_Ri_H29	NA	SAMN02439823	9.3
EURn1S	S_Ri_H05	NA	SAMN02439820	8.2
EURn1S	S_Ri_H23	NA	SAMN02439822	8.5
EURn1S	S_Ri_H43	NA	SAMN02439824	7.9
EURn1S	S_Ri_H07	NA	SAMN02439821	8.4
EURe1	RUS_Ki_H02	NA	SAMEA3334462	14.7
EURe1	RUS_Ki_H03	NA	SAMEA3334455	8.2
EURe1	RUS_Ki_H04	NA	SAMEA3334451	8.9
EURe1	RUS_Tu_H01	NA	SAMEA3334461	16.6
EURe1	RUS_No_H02	NA	SAMEA3334454	9.7
EURe1	RUS_No_H03	NA	SAMEA3334463	19.9
Hyb1	IRL_Lm_H07	NA	SAMEA5089884	14.5
Hyb1	IRL_Lm_H08	NA	SAMEA5089885	17.5
Hyb1	IRL_Lm_H10	NA	SAMEA5089886	22.0

Hyb1	IRL_Lm_H12	NA	SAMEA5089887	15.1
Hyb1	IRL_Lm_H15	NA	SAMEA5089888	15.6
Hyb1	IRL_Lm_H16	NA	SAMEA5089889	13.6
RUS1	RUS_Pr_O01	NA	SAMEA3334604	16.9
RUS1	RUS_Pr_O02	NA	SAMEA3334608	14.5
RUS1	RUS_Pr_O03	NA	SAMEA3334606	15.0
RUS1	RUS_Pr_O04	NA	SAMEA3334605	15.3
RUS1	RUS_Pr_O05	NA	SAMEA3334607	14.3
RUS1	RUS_Ya_O01	NA	SAMEA5089890	10.4
RUS2	RUS_Kr_O01	NA	SAMEA3334610, SAMEA3334615, SAMEA3334594	9.6
RUS2	RUS_Kr_O02	NA	SAMEA3334618	7.6
RUS2	RUS_Kr_O04	NA	SAMEA3334435	9.0
RUS3	RUS_Kr_O03	NA	SAMEA3334617	8.9
RUS3	RUS_Tv_O01	NA	SAMEA3334619	19.9
RUS3	RUS_Tv_O02	NA	SAMEA3334602	16.4
RUS3	RUS_Ya_O02	NA	SAMEA3334603	18.3
RUS3	RUS_Ya_O03	NA	SAMEA3334611	10.4
PEC	CHN_Gu_P01	NA	SAMEA5089891	12.9
PEC	Un_un_P01*	AMNH:261595	SAMEA3334631	7.8
PEC	Un_un_P02	NRM:570709	SAMEA3334629	15.8
Hyb2	RUS_Ke_Y01	NA	SAMEA3334657	9.2
Hyb2	RUS_Ke_Y02	NA	SAMEA3334660	8.7
Hyb2	RUS_Ke_Y03	NA	SAMEA3334658	8.4
Hyb2	RUS_Ke_Y05	NA	SAMEA3334661	19.9
Hyb2	RUS_Ke_Y06	NA	SAMEA3334654	7.2
Outgroup brachyrhynchos	USA_CA_B01	NA	SAMN02743848	NA
Outgroup brachyrhynchos	USA_NJ_B02	NA	SAMN02743849	NA
Outgroup brachyrhynchos	USA_NY_B03	NA	SAMN02743851	NA

Outgroup brachyrhynchos	USA_NY_B04	NA	SAMN02743852	NA
Outgroup brachyrhynchos	USA_CA_B03	NA	SAMN02743850	NA
Outgroup brachyrhynchos	BGI_N302	NA	SAMN02297499	NA
Outgroup moneduloides	NZ_un_N01	NA	SAMEA5816981	NA
Outgroup moneduloides	NZ_un_N02	NA	SAMEA5816982	NA
Outgroup moneduloides	NZ_un_N03	NA	SAMEA5816983	NA
Outgroup moneduloides	NZ_un_N04	NA	SAMEA5816984	NA
Outgroup moneduloides	NZ_un_N05	NA	SAMEA5816976	NA

* Excluded from analysis due to poor quality

Table S2. Summary of the mean genetic diversity and Tajima's D of each population calculated in 50,000bp windows. Population labels are shaded according to plumage color. Populations: Spain (SPA), Germany (EURw1), France (EURw2), Italy (EURs1), Bulgaria and Israel (EURs2), Poland (EURn1P), Sweden (EURn1S), Western Siberia (EURe1), Corse (EURs3), Iraq (IRQ), central Siberia (RUS1), eastern Siberia (RUS2), southeastern Siberia (RUS3), and central Asia (PEC).

Population	pi	Tajima's D
SPA	0.0012	-0.124339
EURw1	0.0013	-1.05168
EURw2	0.0013	-0.914573
EURs1	0.0013	-0.728225
EURs2	0.0013	-0.796137
EURn1P	0.0012	-0.768706
EURn1S	0.0012	-1.0111800
EURe1	0.0013	-0.6084300
EURs3	0.0010	0.5955300
IRQ	0.0011	-0.1127730
RUS1	NA	-0.6871210
RUS2	0.0012	0.5288080
RUS3	0.0014	-0.6225730
PEC	0.0016	-0.2405740

Table S3. Summary of the range of values specified for the estimation of each parameter for the 'genome-wide swamping' scenario, and whether sampling of each parameter uses a log or uniform distribution by fastsimcoal and Jaatha. The est and tpl input file for fastsimcoal, and source code for Jaatha are also accessible on GitHub. Note that the range of values specified for the locus-specific introgression model is the same, but the maximum range for TDIV1, TDIV2 and TEGR2 is specified as TDIV2, TDIV3, and TDIV1, respectively. Note ranges for effective population sizes are expressed as sets of genes (2N) here as required for fastsimcoal, but actually ran as no. of individuals (N) in msprime (i.e. half of the min and max values for Jaatha). Ranges for time are expressed in generations.

**Parameters**	**Fastsimcoal**			**Jaatha**		
	**Distribution**	**Min**	**Max**	**Distribution**	**Min**	**Max**
NANC1	log	1E+03	1E+06	log	2E+03	1E+06
NANC2	log	1E+03	1E+06	log	2E+03	1E+06
NANC3	log	1E+03	1E+06	log	2E+03	1E+06
NPOP1	log	1E+03	1E+06	log	2E+03	1E+06
NPOP2	log	1E+03	1E+06	log	2E+03	1E+06
NPOP3	log	1E+03	1E+06	log	2E+03	1E+06
NPOP4	log	1E+03	1E+06	log	2E+03	1E+06
TDIV3	log	TMRMG	1E+06	NA	NA	NA
TDIV2	log	TMRMG	1E+06	NA	NA	NA
TDIV1	log	TMRMG	1E+06	NA	NA	NA
TMRMG	log	1E+02	1E+04	log	100	10000
TEGR1	log	1	TMRMG	NA	NA	NA
TEGR2	log	TMRMG	TDIV2	NA	NA	NA
GROW1	uniform	-1E-03	0	uniform	0	-0.0001
GROW2	uniform	-1E-03	0	uniform	0	-0.0001
MIG01R	uniform	5E-06	1E-02	uniform	0	0.001
MIG10R	uniform	5E-06	1E-02	uniform	0	0.001
MIG12R	uniform	5E-06	1E-02	uniform	0	0.001
MIG21R	uniform	5E-06	1E-02	uniform	0	0.001
MIG32R	uniform	5E-06	1E-02	uniform	0	0.001
recom rate	NA	NA	NA	Uniform	1.06E-09	9.54E-08

TDIV2-TDIV1	NA	NA	NA	log	100	100000
TDIV1-TMRMG	NA	NA	NA	log	100	100000
TEGR1/TDIV1	NA	NA	NA	uniform	0	1
TEGR2/TDIV2	NA	NA	NA	uniform	0	1

Table S4. Matrix of the mean genetic differentiation (F_{ST}) of all possible pairwise comparisons calculated in 50,000bp windows. High F_{ST} values above 0.9 are highlighted in bold. Population labels are shaded according to plumage color (black, grey or pied). Populations: Spain (SPA), Germany (EURw1), France (EURw2), Italy (EURs1), Bulgaria and Israel (EURs2), Poland (EURn1P), Sweden (EURn1S), Western Siberia (EURe1), Corse (EURs3), Iraq (IRQ), central Siberia (RUS1), eastern Siberia (RUS2), southeastern Siberia (RUS3), and central Asia (PEC).

	SPA	EURw1	EURw2	EURs1	EURs2	EURn1P	EURn1S	EURe1	EURs3	IRQ	RUS1	RUS2	RUS3	PEC
SPA														
EURw1	0.0468													
EURw2	0.0346	0.0036												
EURs1	0.0751	0.0133	0.0174											
EURs2	0.0715	0.0115	0.0173	0.0095										
EURn1P	0.0743	0.0119	0.0160	0.0124	0.0098									
EURn1S	0.0699	0.0078	0.0121	0.0090	0.0070	0.0070								
EURe1	0.0626	0.0102	0.0166	0.0112	0.0112	0.0095	0.0053							
EURs3	0.0700	0.0343	0.0484	0.0357	0.0447	0.0370	0.0342	0.0596						
IRQ	**0.0903**	0.0511	0.0636	0.0566	0.0589	0.0559	0.0519	0.0720	**0.1269**					
RUS1	0.0660	0.0212	0.0278	0.0259	0.0265	0.0234	0.0184	0.0216	NA	0.0781				
RUS2	0.0800	0.0496	0.0672	0.0599	0.0660	0.0540	0.0498	0.0776	NA	**0.1366**	0.0552			
RUS3	0.0719	0.0419	0.0502	0.0510	0.0549	0.0485	0.0441	0.0556	NA	**0.0984**	0.0341	0.0687		
PEC	0.0456	0.0284	0.0392	0.0351	0.0420	0.0331	0.0305	0.0528	NA	**0.0940**	0.0371	0.0876	0.0153	

Table S5. Original and bootstrap corrected (Bscor) Jaatha's estimates with the lower and upper boundary for confidence interval. Time is presented in generations (without converting to years) and effective population size is presented as sets of gene (2n for diploid). See **Figure S3** for parameter definition.

Parameter	Original	Bscor	Lower (2.5%)	Upper (97.5%)
NANC1	9.41E+05	9.50E+05	9.41E+05	10.3E+05
NANC2	9.41E+05	9.85E+05	9.41E+05	12.5E+05
NANC3	8.78E+04	8.89E+04	8.74E+04	9.02E+04
NPOP1	1.65E+04	2.14E+04	1.81E+04	2.57E+04
NPOP2	7.50E+04	9.96E+04	8.26E+04	1.20E+05
NPOP3	1.11E+05	1.36E+05	1.17E+05	1.59E+05
NPOP4	3.77E+04	4.35E+04	3.98E+04	4.68E+04
TDIV3	7.39E+04	7.67E+04	7.00E+04	8.63E+04
TDIV2	1.84E+04	2.45E+04	2.04E+04	3.24E+04
TDIV1	5.39E+03	7.32E+03	5.91E+03	9.16E+03
TMRMG	5.29E+03	7.26E+03	5.89E+03	9.05E+03
TEGR1	4.84E+03	8.39E+03	4.88E+03	1.57E+04
TEGR2	3.08E+03	2.08E+02	-9.44E+03	1.04E+04
GROW1	-3.74E-05	-5.17E-05	2.205E-06	-7.33E-05
GROW2	-8.74E-05	-1.23E-04	-8.08E-05	-1.719E--04
MIG01R	9.52E-05	6.14E-05	3.63E-05	8.27E-05
MIG10R	9.70E-05	6.57E-05	4.06E-05	8.64E-05
MIG12R	2.42E-04	1.55E-04	9.02E-05	2.11E-04
MIG21R	9.32E-05	6.83E-05	4.97E-05	8.34E-05
MIG23R	8.82E-06	8.79E-06	8.78E-06	8.82E-06
MIG32R	4.41E-05	3.39E-05	2.97E-05	3.91E-05
recom rate	1.09E-09	1.09E-09	1.02E-09	1.10E-09

Table S6. Original and bootstrap corrected (Bscor) fastsimcoal's estimates with the lower and upper boundary for confidence interval. Time is presented in generations (without converting to years) and effective population size is presented as sets of gene (2n for diploid). See **Figure S3** for parameter definition.

Parameter	Original	Bscor	Lower (2.5%)	Upper (97.5%)
NANC1	1.13E+06	1.42E+06	1.72E+05	1.14E+06
NANC2	1.08E+06	1.38E+06	1.67E+05	1.14E+06
NANC3	7.63E+04	4.93E+04	6.72E+04	4.07E+05
NPOP1	1.00E+04	-6.51E+02	1.22E+03	5.50E+04
NPOP2	2.74E+05	1.65E+05	5.31E+04	8.33E+05
NPOP3	4.67E+05	5.79E+05	5.44E+03	8.74E+05
NPOP4	3.56E+04	4.30E+04	1.39E+03	5.35E+04
TDIV3	8.70E+04	9.46E+04	6.31E+04	1.14E+05
TDIV2	2.27E+04	2.92E+04	1.83E+03	4.29E+04
TDIV1	5.96E+03	-2.26E+03	5.11E+02	3.55E+04
TMRMG	3.46E+03	2.71E+03	1.96E+02	8.76E+03
TEGR1	5.36E+02	6.27E+03	2.57E+02	1.49E+04
TEGR2	5.96E+03	-1.82E+01	2.28E+00	5.33E+03
GROW1	-2.35E-04	-1.38E-04	-9.59E-04	-1.00E-04
GROW2	-2.54E-04	-1.19E-04	-9.80E-04	-3.79E-05
MIG01R	1.69E-04	-2.94E-06	2.74E-05	1.76E-03
MIG10R	3.16E-04	1.60E0-4	7.99E-06	2.67E-03
MIG12R	6.94E-04	4.36E-05	8.94E-06	7.12E-03
MIG21R	1.29E-04	-5.71E-04	3.46E-05	3.96E-03
MIG23R	5.68E-06	-3.28E-05	5.70E-06	2.70E-04
MIG32R	4.52E-05	-1.23E-04	1.72E-05	1.37E-03

Table S7. A list of genes found in the underestimated regions identified from the model fit of joint-site frequency spetra (j-SFS) of simulated and empirical data (see **Figure S11-S13**, blue areas in j-SFS of EURw and EURns). A total of 293 underestimated sites were identified, primarily from scaffold 7 (chr8 (NCBI ID:NC_046338.1); 161 sites spanning across ~150,000bp from position 58,259 to 205,720) and scaffold 29 (chr15 (NCBI ID: NC_046345.1); 73 sites spanning across ~335,700bp from position 9,591,051 to 9,926,684).

**Scaffold**	**Chromosome**	**Gene**	**Ensembe ID**
29	15	BCR	ENSCCCG00025006110
29	15	GNAZ	ENSCCCG00025006219
29	15	RSPH14	ENSCCCG00025006208
29	15	RAB36	ENSCCCG00025006186
29	15	YWHAH	ENSCCCG00025006270
29	15	SLC54A1	ENSCCCG00025006302
29	15	ANHX	ENSCCCG00025006554
29	15	DEPDC5	ENSCCCG00025006566
7	8	SLC44A5	ENSCCCG00025006566
7	8	LHX8	ENSCCCG00025009754

Table S8. Time of divergence (in years) between all-black carrion and gray-coated hooded crows estimated with mean absolute genetic differentiation (d_a) of each pairwise comparison from either chromosome 18 or the putatively neutral region. Time of divergence was computed with the following formula: Time of divergence = Mean(d_a) / 2 * Mutation rate (=3.18e-09) * Generation time (=5.79). All-black carrion crows represented by populations from Spain (SPA), Germany (EURw1) and France (EURw2). Gray-coated crows represented by populations from Iraq (IRQ) and Poland (EURn1P).

**Pairwise comparison**	**chr18**		**Neutral**	
	**IRQ**	**EURn1P**	**IRQ**	**EURn1P**
**SPA**	343,000	316,000	255,000	159,000
**EURw1**	373,000	352,000	148,000	22,600
**EURw2**	317,000	306,000	151,000	34,900

References

Baumdicker, F., Bisschop, G., Goldstein, D., Gower, G., Ragsdale, A. P., Tsambos, G., Zhu, S., Eldon, B., Ellerman, E. C., Galloway, J. G., Gladstein, A. L., Gorjanc, G., Guo, B., Jeffery, B., Kretzschumar, W. W., Lohse, K., Matschiner, M., Nelson, D., Pope, N. S., … Kelleher, J. (2022). Efficient ancestry and mutation simulation with msprime 1.0. *Genetics*, *220*(3), iyab229. https://doi.org/10.1093/genetics/iyab229

Bozdogan, H. (1987). Model selection and Akaike's Information Criterion (AIC): The general theory and its analytical extensions. *Psychometrika*, *52*(3), 345–370. https://doi.org/10.1007/BF02294361

Danecek, P., Auton, A., Abecasis, G., Albers, C. A., Banks, E., DePristo, M. A., Handsaker, R. E., Lunter, G., Marth, G. T., Sherry, S. T., McVean, G., Durbin, R., & Group, 1000 Genomes Project Analysis. (2011). The variant call format and VCFtools. *Bioinformatics*, *27*(15), 2156–2158. https://doi.org/10.1093/bioinformatics/btr330

Danecek, P., Bonfield, J. K., Liddle, J., Marshall, J., Ohan, V., Pollard, M. O., Whitwham, A., Keane, T., McCarthy, S. A., Davies, R. M., & Li, H. (2021). Twelve years of SAMtools and BCFtools. *GigaScience*, *10*(2), giab008. https://doi.org/10.1093/gigascience/giab008

Efron, B., & Tibshirani, R. J. (1994). *An introduction to the bootstrap*. Chapman and Hall/CRC. https://doi.org/10.1201/9780429246593

Excoffier, L., Marchi, N., Marques, D. A., Matthey-Doret, R., Gouy, A., & Sousa, V. C. (2021). *fastsimcoal2*: Demographic inference under complex evolutionary scenarios. *Bioinformatics*, *37*(24), 4882–4885. https://doi.org/10.1093/bioinformatics/btab468

Gutenkunst, R. N., Hernandez, R. D., Williamson, S. H., & Bustamante, C. D. (2009). Inferring the joint demographic history of multiple populations from multidimensional SNP frequency data. *PLOS Genetics*, *5*(10), e1000695. https://doi.org/10.1371/journal.pgen.1000695

Hasegawa, M., Kishino, H., & Yano, T. (1985). Dating of the human-ape splitting by a molecular clock of mitochondrial DNA. *Journal of Molecular Evolution*, *22*(2), 160–174. https://doi.org/10.1007/BF02101694

Hudson, R. R., & Kaplan, N. L. (1985). Statistical properties of the number of recombination events in the history of a sample of DNA sequences. *Genetics*, *111*(1), 147–164. https://doi.org/10.1093/genetics/111.1.147

Korneliussen, T. S., Albrechtsen, A., & Nielsen, R. (2014). ANGSD: Analysis of next generation sequencing data. *BMC Bioinformatics*, *15*(1), 356. https://doi.org/10.1186/s12859-014-0356-4

Mathew, L. A., Staab, P. R., Rose, L. E., & Metzler, D. (2013). Why to account for finite sites in population genetic studies and how to do this with Jaatha 2.0. *Ecology and Evolution*, *3*(11), 3647–3662. https://doi.org/10.1002/ece3.722

McKenna, A., Hanna, M., Banks, E., Sivachenko, A., Cibulskis, K., Kernytsky, A., Garimella, K., Altshuler, D., Gabriel, S., Daly, M., & DePristo, M. A. (2010). The Genome Analysis Toolkit: A MapReduce framework for analyzing next-generation DNA sequencing data. *Genome Research*, *20*(9), 1297–1303. https://doi.org/10.1101/gr.107524.110

Meng, X. L., & Rubin, D. B. (1993). Maximum likelihood estimation via the ECM algorithm: A general framework. *Biometrika*, *80*(2), 267–278. https://doi.org/10.1093/biomet/80.2.267

Naduvilezhath, L., Rose, L. E., & Metzler, D. (2011). Jaatha: A fast composite-likelihood approach to estimate demographic parameters: estimation of demographic parameters. *Molecular Ecology*, *20*(13), 2709–2723. https://doi.org/10.1111/j.1365-294X.2011.05131.x

Rambaut, A., & Grassly, N. C. (1997). Seq-Gen: An application for the Monte Carlo simulation of DNA sequence evolution along phylogenetic trees. *Bioinformatics*, *13*(3), 235–238. https://doi.org/10.1093/bioinformatics/13.3.235

Staab, P. R., Zhu, S., Metzler, D., & Lunter, G. (2015). scrm: Efficiently simulating long sequences using the approximated coalescent with recombination. *Bioinformatics*, *31*(10), 1680–1682. https://doi.org/10.1093/bioinformatics/btu861
